# Importance of Chiral Recognition in Designing Metal-Free Ligands for G-Quadruplex DNA

**DOI:** 10.3390/molecules24081473

**Published:** 2019-04-15

**Authors:** Dora M. Răsădean, Samuel W. O. Harrison, Isobel R. Owens, Aucéanne Miramont, Frances M. Bromley, G. Dan Pantoș

**Affiliations:** Department of Chemistry, University of Bath, Claverton Down, Bath BA2 7AY, UK; D.Rasadean@bath.ac.uk (D.M.R.); swoharrison@gmail.com (S.W.O.H.); isobelowens1@gmail.com (I.R.O.); a.miramont@escom.fr (A.M.); francesbromley95@gmail.com (F.M.B.)

**Keywords:** circular dichroism, G-quadruplex DNA, chirality, enantioselectivity, chiral recognition

## Abstract

Four pairs of amino acid-functionalized naphthalenediimide enantiomers (d- and l-lysine derived NDIs) were screened toward G-quadruplex forming sequences in telomeres (h-TELO) and oncogene promoters: c-KIT1, c-KIT2, k-RAS and BCL-2. This is the first study to address the effect of point chirality toward G-quadruplex DNA stabilization using purely small organic molecules. Enantioselective behavior toward the majority of ligands was observed, particularly in the case of parallel conformations of c-KIT2 and k-RAS. Additionally, *N*_ε_-Boc-l-Lys-NDI and *N*_ε_-Boc-d-Lys-NDI discriminate between quadruplexes with parallel and hybrid topologies, which has not previously been observed with enantiomeric ligands.

## 1. Introduction

**Figure 1:**
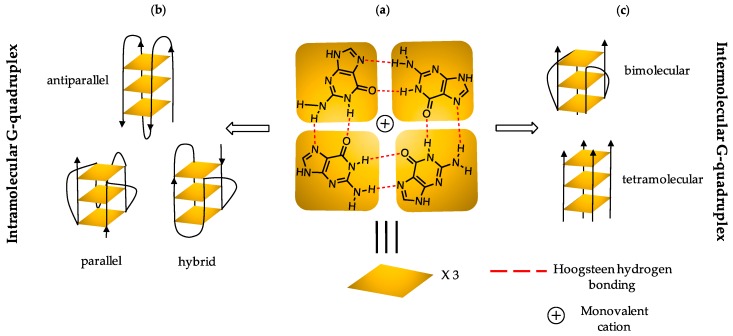
(**a**) Schematic representation of the structural unit of G-quadruplex DNA held together by a cyclic Hoogsteen hydrogen bonding network stabilized by monovalent cations; cartoon representations of: (**b**) intramolecular and (**c**) intermolecular topologies adopted by G-quadruplex DNA (G4 DNA). Arrow direction is from 5′ to 3′.

The demand for alternative approaches to the classical chemotherapy as principal treatment of cancer has led to the development of advanced techniques, and to the identification of new targets [1]. Among these, G-quadruplex DNA (G4 DNA) has emerged as a promising target as it is over-expressed in the promoter regions of a wide number of oncogenes as well as telomeres [2,3,4]. DNA sequences that are sufficiently rich in guanine (G) have the ability to self-assemble into four stranded G-quadruplex (G4) structures [4]. The main structural component of G4 DNA is a planar arrangement of four guanines held together by cyclic Hoogsteen hydrogen bonding networks [5] (Figure 1). The directionality of the strands defines the folding topology as parallel, antiparallel, or a hybrid conformation [6], as illustrated in Figure 1. The type of conformation adopted by sequences is driven by a monovalent cation, which co-ordinates to the O6 oxygen of each G and thus stabilizes the G4 [4,7,8]. G4 DNA governs several vital biological processes such as transcription, translation and replication [9,10], which makes it a promising target for anticancer therapies.

There is direct evidence that small ligands can suppress gene transcription, and therefore induce apoptosis [11]. However, the structural and topological diversity of quadruplexes and ligand-binding specificity remain key challenges in developing effective and selective G4 DNA-binding ligands [12]. A considerable number of small ligands have been developed as potential G4 binders, including Telomestatin^®^, porphyrinoids, quinolones, alkaloids, carbazoles, acridine derivatives [13,14,15,16,17,18,19,20,21,22,23,24,25,26], and recently, reductive-activated G4-binders [27]. Besides ligands that have a planar surface for interaction with G4 DNA, molecules containing bi-aryl linkages have also emerged recently as promising candidates [28,29,30,31]. Representative examples include: phenanthroline-bisoxazoles [31], pentaheteroaryls [28], oxazoles [29], and oligomeric pyridyl-oxazoles [30] ligands. However, only one candidate—Quarfloxin^®^—has advanced to Phase II clinical trials albeit unsuccessfully [32].

**Figure 2:**
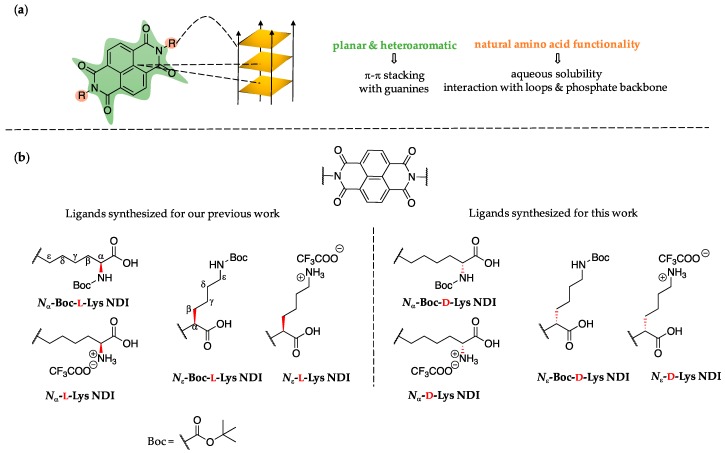
(**a**) Structural features of naphthalenediimide (NDI) molecules synthesized and interaction with G4 DNA; (**b**) chemical structures of l-NDIs (left) and d-NDIs (right) used in our studies and their corresponding names. The naming is based on the position of the carboxylic acid relative to the NDI core: either distal (*N*_α_-Lys NDIs) or proximal (*N*_ε_-Lys NDIs). The l or d chirality is highlighted in red.

A scaffold that has attracted interest as potential G4 DNA-binding ligand is naphthalenediimide (NDI). NDI-based molecules consist of a large, planar and heteroaromatic surface that can interact with G4 DNA via aromatic π-π stacking [33,34]. The literature reports di-, tri-, and tetra-substituted NDI ligands with high affinity for G4 [8,9,35,36,37,38,39,40,41]. We have recently reported the synthesis of symmetrical di-substituted NDIs bearing amino acids as peripheral substituents [35]. One of the ligands (*N*_ε_-Boc-l-Lys NDI, see Figure 2) has exhibited a highly discriminating nature by stabilizing only the oncogene promoter c-KIT2 [35].

One of the key features of the series of NDIs we have previously reported is their chiral nature, which was not discussed in detail in our previous study. Chirality is ubiquitous in nature and plays crucial roles in drug design [42,43], thus we decided to explore the influence of chirality on the interaction of our NDI-based ligands with G4 DNA. It is widely known that enantiomers can display different biological activity on the basis of toxicology, pharmacology, pharmacokinetics and metabolism [42]. To the best of our knowledge, all chiral G4-ligands explored so far are metal-containing, and can be categorized into either metallo-supramolecular assemblies [44,45,46] or complexes with transition metals (Ru, Cu, Ni, Fe, Zn, Pt) [47,48,49,50,51,52,53,54,55,56,57]. A recently reported study on metallohelices and enantiomeric G-quadruplex DNA showed an interesting mirror-image dependence of ligands binding to l- or d-DNA [58]. Although point chirality is present in Telomestatin^®^ (a natural product) and Quarfloxin^®^ [32,51,59], no account or consideration of the significance of point chirality/enantiomeric forms is given in the literature.

The current study comes as a complementary work on the series of l-NDIs we have recently reported [35], thus references to this will be made throughout this manuscript. This work highlights the significance of point chirality toward stabilization of G4 within telomeres (h-TELO) and oncogene promoter regions k-RAS, c-KIT and BCL-2. The k-RAS sequence is dis-regulated in pancreatic cancer [9], while c-KIT2 is upregulated up to 80% in breast malignancies [60]. BCL-2 is highly expressed in androgen-independent tumors in advanced states of prostate cancer [61,62]. BCL-2 is also dis-regulated in pancreatic cancer; therefore, BCL-2 has emerged as an important target for both prostate and pancreatic cancers therapies [61,62]. We have changed the chirality from l- to d-enantiomers of lysine-functionalized NDIs and have explored their potential as G4 DNA as well as dsDNA binders. The interaction with the latter must be very small so that any G4 DNA potential ligands do not affect the dsDNA present in healthy cells. This work paves the way for understanding the importance of chiral recognition in designing efficient ligands for G4 DNA.

## 2. Results and Discussion

### 2.1. Ligand Design

The NDI scaffold is an attractive platform for designing G4 ligands due to the electron-deficient polyaromatic core that can stack on the guanine tetrads. We have chosen lysine (Lys) due to its ideal structural features to interacting with G-quadruplex DNA. Lys functionalization imparts water solubility and positive charges that can interact with the DNA’s phosphate backbone. Our previous work indicated that the length of peripheral substituents played a role in binding [35]. Lys showed best binding (and selective behavior) for certain quadruplexes [35]. The key features that make these NDIs potential G4 DNA binders are highlighted in Figure 2a.

This study is focused on the influence of chirality on the G4-ligand interaction. The chemical structures of the Lys-NDI derivatives are provided in Figure 2b. The ligands can be categorized depending on: (1) Lys connectivity, where α- corresponds to the stereocentre distal from the NDI core, while ε- represents a structure with the stereocentre proximal to the NDI core; and (2) presence or absence of the *N*-Boc protecting group of the amine functionality. The synthetic route assumed a one-step microwave-assisted reaction (for Boc-protected NDIs), followed by a classic deprotection with trifluoroacetic acid (TFA) to yield the deprotected derivatives [35,63].

All experiments were performed in PBS (phosphate-buffered saline, 10 mM) containing 100 mM of potassium fluoride at pH 7.4 (as source of cations) in order to resemble the physiological conditions. Although slightly lower than generally found in cells [64], the K^+^ concentration provides the fundamental cation channel for quadruplex formation.

### 2.2. G4 DNA Topology Analysis

G4 DNA is known to adopt different conformations depending on the conditions in which the annealing is performed. The folding topologies of the sequences used in this work were determined by circular dichroism (CD) analysis (CD spectra of all sequences are provided in Supplementary Material, Figure S1), and are in agreement with previously reported information [35]. The CD profile of k-RAS and c-KIT2 in PBS at pH 7.4 displayed characteristics of a parallel topology, with large positive Cotton effects around 260 nm, and smaller negative peaks at shorter wavelengths. The folding of k-RAS and c-KIT2 into parallel conformations is consistent with other studies reported in the literature [6,65,66,67,68]. The only telomeric sequence in this study—h-TELO—folded in a hybrid manner, and its CD fingerprint was characterized by a positive peak at 290 nm and a small negative peak at 260 nm. Previously reported studies also confirm the hybrid arrangement of h-TELO forming-quadruplex DNA [69,70,71,72,73,74]. The CD spectra of c-KIT1 and BCL-2 exhibited spectral features corresponding to parallel topologies. However, they showed small and positive shoulder peaks around 290 nm, indicating the hybrid character of these two sequences under these particular experimental conditions (3:1 parallel to antiparallel ratio) (see Supplementary Material, Figure S1d). c-KIT1 has been previously reported as folding into mixed parallel:antiparallel conformation under K^+^ conditions [75]. BCL-2 sequence can form either a hybrid or a 1:13:1 parallel structure, depending on the length and type of nucleobases [76,77,78,79,80,81,82,83,84]. The BCL-2 fragment used in this study folds into a hybrid structure, as confirmed by CD studies. The only non-quadruplex forming sequence—dsDNA—was characterized by a positive peak at 272 nm and a large negative peak around 250 nm. Quadruplex topology was retained on addition of ligand; however, it was accompanied by a minor shift of 1–3 nm and reduced peak intensity [31]. The G-quadruplex sequences do not change their conformation upon NDIs binding, as indicated by CD spectra before (Figures S3–S9) and during (Figure S10) variable-temperature (VT) CD studies (without and with ligands; further details in Supplementary Materials). To further show this, CD titration studies of increasing equivalents of ligand into DNA were performed (Figure S11).

### 2.3. CD Thermal Melting Studies

The stabilization effect of the NDI ligands toward a panel of G4 DNA sequences (k-RAS, c-KIT1, c-KIT2, BCL-2, h-TELO) as well as dsDNA was investigated by performing VT CD studies. The melting, or denaturation, of DNA is characterized by the loss of CD response, and the melting temperature (*T*_m_, reported in °C) is defined as the equilibrium state at which 50% of the DNA is folded and 50% unfolded. The profile of a CD melting curve gives information on the progress of DNA denaturation as temperature is increased. The *T*_m_ of G4 DNA-NDI complexes is directly linked with the number of available binding sites, binding fashion and the affinity of each ligand for a particular topology. DNA can melt in either a single-step denaturation, showing isosbestic points, or a concerted process, in which multiple stable intermediates can be observed.

The VT CD experiment design involved the addition of 10 equivalents of ligand to the DNA sequence solution and sequential CD data acquisition over a 5–95 °C temperature range. The Boc-protected NDI ligands are soluble in aqueous solution, while the deprotected ones are prone to aggregation. Therefore, solutions of all ligands in PBS were prepared at concentrations of 300 μM, except for *N*_ε_-d-Lys NDI, concentration of which was 75 μM. The reported melting temperatures of DNA interacting with *N*_ε_-Lys NDI represent a combination of the temperature induced de-aggregation of *N*_ε_-Lys NDI and the ligand-DNA binding processes.

The melting profile of each G4 DNA-NDI complex was monitored at a single-wavelength, allowing a larger number of data points to be obtained in the same (or shorter) timeframe compared to measuring across the entire wavelength range. The relevant single-wavelength for VT CD analysis was selected by acquiring a CD spectrum across the wavelength range 220–420 nm; the wavelength corresponding to the DNA peak with maximum ellipticity (λ_max_) was chosen. The data was fitted using the Boltzmann mathematical model; the *T*_m_ was determined as the midpoint of the sigmoidal melting curve. The stabilization effect of the NDIs toward the DNA sequences is defined by the difference in melting temperature (Δ*T*_m_, also reported in °C) between the ligand/DNA complex and the DNA by itself. A positive Δ*T*_m_ indicates that the melting temperature has increased, and thus the ligand has stabilized the DNA sequence under the experimental conditions used. 

The results are discussed in terms of mean Δ*T*_m_ and in conjunction with those obtained from the l-NDI series of ligands [35]. This allows comparison between the stabilization effect of the pair of enantiomers (i.e., α- and ε-connectivities as well as protected/deprotected versions for both l- and d-isomers) toward G4 DNA-forming sequences and dsDNA. The errors of *T*_m_ values in the case of l-NDIs are larger compared to those from d-ligands because of different number of data points obtained in each experiment: ~19 for l-NDIs and ~90 for d-NDIs. This affects mainly the sequences exhibiting high *T*_m_ such as c-KIT1 and c-KIT2. However, the data obtained for l- vs. d- will be used to illustrate the direction the ligand influences the DNA (i.e., stabilisation/destabilisation). In the case of BCL-2, both l-Lys and d-Lys NDI derivatives have been investigated, as the BCL-2 sequence was not part of our original screen. 

#### 2.3.1. c-KIT2

**Figure 3:**
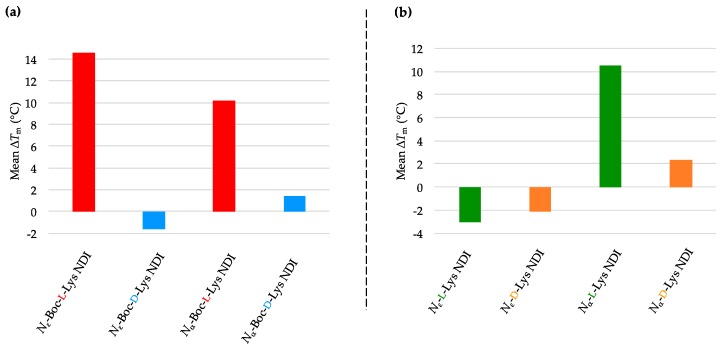
Graphical representations of mean Δ*T*_m_ values obtained from variable-temperature circular dichroism (VT CD) studies of c-KIT2 with 10 equivalents of (**a**) *N*_α_- and *N*_ε_-Boc-l- and d-NDIs as well as (**b**) *N*_α_- and *N*_ε_-l- and d-NDIs.

The *N*_ε_-Boc-l-Lys NDI stabilizes c-KIT2 by almost 15 °C, while a 10 °C stabilization was observed for its α-connectivity counterpart (*N*_α_-Boc-l-Lys NDI) [35]. Remarkably, their enantiomers, *N*_ε_-Boc-d-Lys NDI and *N*_α_-Boc-d-Lys NDI, have very little interaction with c-KIT2 (Δ*T*_m_ = −1.6 and +1.4 °C, respectively; Figure 3a and Table 1a. This finding is very important as it shows, for the first time, that enantiomeric ligands have different strength of interaction with a quadruplex DNA, namely c-KIT2. Therefore, the difference in point chirality between the two ligands imposes a “matched” interaction with the binding site of c-KIT2 in the case of l-NDI, whereas the interaction with the opposite three-dimensional structure is not favorable. The larger difference in stabilization between the ε- enantiomers vs. α- enantiomers emphasizes that the closer the point chirality to the NDI core, the higher selectivity in binding is observed. In the case of ε-connectivity derivatives, the chiral center is close to the NDI core (thus the selective interaction), while the α-NDIs have the chiral center five bonds away from the core. The ε-deprotected versions of both enantiomers exhibited destabilization toward c-KIT2, while the NDIs with α-connectivity showed favorable, but less selective interaction with its structure (Figure 3b and Table 1b). The VT CD single-wavelength spectra of c-KIT2 by itself as well as c-KIT2-NDI assemblies and their corresponding Boltzmann fitting curves are illustrated in Supplementary Material, Figure S12.

#### 2.3.2. c-KIT1

**Figure 4:**
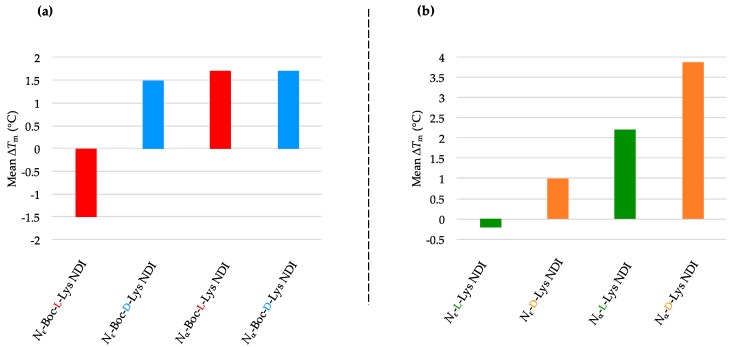
Graphical representations of mean Δ*T*_m_ values obtained from VT CD studies of c-KIT1 with 10 equivalents of (**a**) *N*_α_- and *N*_ε_-Boc-l- and d-NDIs as well as (**b**) *N*_α_- and *N*_ε_- l- and d-NDIs.

Both enantiomers of *N*_ε_-Boc-Lys NDI ligands discriminate between c-KIT1 and c-KIT2, even though they are putative G4-forming sequences within the same promoter. The pair *N*_ε_-Boc-l-Lys/*N*_ε_-Boc-d-Lys NDIs displayed an opposite stabilization behavior toward c-KIT1 compared to c-KIT2. While *N*_ε_-Boc-l-Lys NDI destabilized the sequence by −1.5 °C, its enantiomer induced a minor stabilization of 1.5 °C. Both α-protected enantiomers exhibited small stabilization of c-KIT1 hybrid structure. As in the case of c-KIT2, chiral recognition of the ε-connectivity ligands was observed. All deprotected NDIs show minor stabilization (<2 °C), indicating that the ligands interact weakly with the DNA. The mean Δ*T*_m_ obtained from VT CD studies of c-KIT1 with NDI ligands are graphically represented in Figure 4a,b, and the *T*_m_ values of c-KIT1 as well as c-KIT1-NDI assemblies are provided in Table 2a,b. The VT CD single-wavelength spectra of c-KIT1 by itself as well as c-KIT1-NDI assemblies and their corresponding Boltzmann fitting curves are shown in Supplementary Material, Figure S13.

#### 2.3.3. k-RAS

The oncogene promoter k-RAS adopts, under our experimental conditions, a parallel conformation with short loops. It has the lowest melting temperature of all DNA sequences used in this study. The melting profiles of k-RAS displayed characteristic sigmoidal curves, regardless of the ligand used. Good fitting of the Boltzmann mathematical model resulted melting studies with the lowest *T*_m_ errors. All Boc-protected ligands showed discriminating behavior toward k-RAS. Both *N*_ε_-Boc-l-Lys and *N*_α_-Boc-l-Lys NDIs induced a minor stabilization on this sequence (2 °C), while their enantiomers destabilized it. The position of the chiral center, proximal or distal, with respect to the NDI has little influence on the interaction with k-RAS, whereas the chirality of the stereocenter is the determining factor in the interaction between this quadruplex and the NDI ligands. 

**Figure 5:**
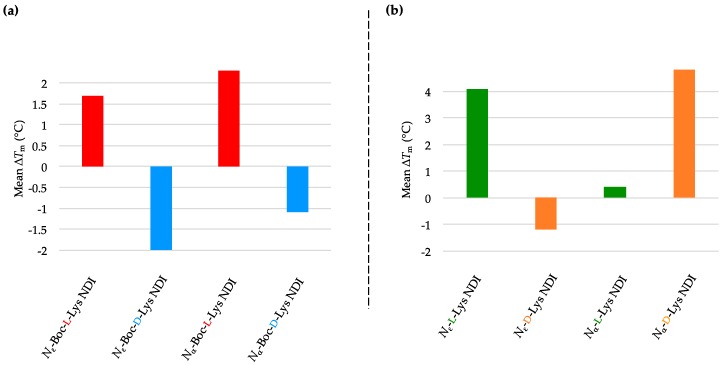
Graphical representations of mean Δ*T*_m_ values obtained from VT CD studies of k-RAS with 10 equivalents of (**a**) *N*_α_- and *N*_ε_-Boc-l- and d-NDIs as well as (**b**) *N*_α_- and *N*_ε_-l- and d-NDIs.

Similar behavior was observed for the deprotected ligands, with *N*_ε_-l-Lys NDI moderately stabilizing k-RAS, and its enantiomer destabilizing it. The d-enantiomer of α-NDI also has a stabilizing effect, but as this ligand is prone to aggregation, these results should be considered as a combination between ligand de-aggregation and interaction with the DNA. The results are provided as graphs in Figure 5a,b, and the *T*_m_ values of k-RAS as well as k-RAS-NDI assemblies are summarized in Table 3a,b. The VT CD single-wavelength spectra of k-RAS by itself as well as k-RAS-NDI assemblies and their corresponding Boltzmann fitting curves are depicted in Supplementary Material, Figure S14.

#### 2.3.4. BCL-2

**Figure 6:**
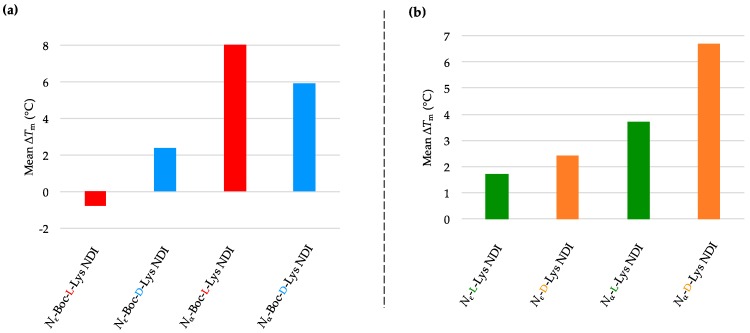
Graphical representations of mean Δ*T*_m_ values obtained from VT CD studies of BCL-2 with 10 equivalents of (**a**) *N*_α_- and *N*_ε_-Boc-l- and d-NDIs as well as (**b**) *N*_α_- and *N*_ε_-l- and d-NDIs.

The Boc-protected NDIs stabilized the BCL-2 sequence, except *N*_ε_-Boc-l-Lys NDI, which showed minor destabilization of this sequence, as shown in Figure 6a and Table 4a. The best stabilization in the ligand series was induced by *N*_α_-Boc-l-Lys NDI, with a Δ*T*_m_ of 9 °C. This sequence exhibits enantioselective interaction with the enantiomers with ε-connectivity (chiral center close the NDI unit): *N*_ε_-Boc-l-Lys NDI stabilizes it, whereas its enantiomer does not. All the deprotected ligands have a stabilizing effect on BCL-2, with no selectivity l- vs. d-enantiomers, or α- vs. ε-connectivities (Figure 6b and Table 4b). The VT CD single-wavelength spectra of BCL-2 by itself as well as BCL-2-NDI assemblies and their corresponding Boltzmann fitting curves are given in Material: Supplementary Material: Figures S15 (for l-NDIs) and S16 (for d-NDIs).

#### 2.3.5. h-TELO

**Figure 7:**
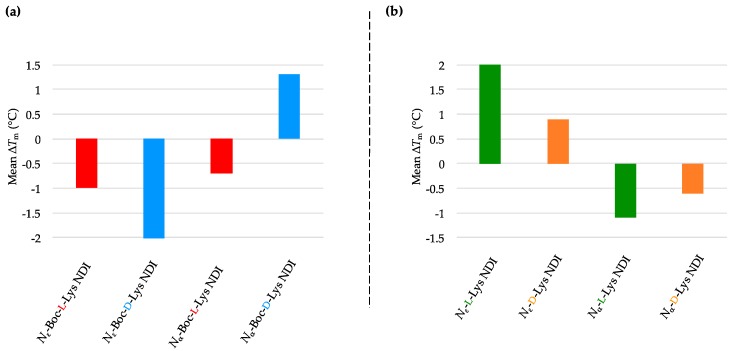
Graphical representations of mean Δ*T*_m_ values obtained from VT CD studies of h-TELO with 10 equivalents of (**a**) *N*_α_- and *N*_ε_-Boc-l- and d-NDIs as well as (**b**) *N*_α_- and *N*_ε_-l- and d-NDIs.

The hybrid conformation h-TELO interacts weakly with the majority of NDI ligands studied. This suggests that this topology is poorly and non-selectively stabilized by Lys-functionalized di-substituted NDIs, which is consistent with the results obtained for also hybrid c-KIT1 and BCL-2 (except the ε-connectivity ligands). The results are graphically represented as mean Δ*T*_m_ in Figure 7a,b. The *T*_m_ values of h-TELO as well as h-TELO-NDI assemblies are listed in Table 5a,b. The VT CD single-wavelength spectra of h-TELO by itself as well as h-TELO-NDI assemblies and their corresponding Boltzmann fitting curves are provided in Supplementary Material, Figure S17.

#### 2.3.6. dsDNA

*N*_α_-d-Lys NDI largely destabilized dsDNA by around 5 °C, while *N*_α_-Boc-l-Lys and *N*_ε_-l-Lys NDIs displayed minor stabilization effects (Δ*T*_m_ < 2 °C), as depicted in Table 6a,b. This indicates that such types of ligands have selectivity for certain G4 DNA over dsDNA. The VT CD single-wavelength spectra of dsDNA by itself as well as dsDNA-NDI assemblies and their corresponding Boltzmann fitting curves are given in Supplementary Material, Figure S18.

## 3. Materials and Methods

The oligonucleotides used in this study were purchased from Invitrogen^®^, having the following sequences (from 5′ to 3′): c-KIT1: GGG AGG GCG CTG GGA GGA GGG; c-KIT2: GGG CGG GCG CGA GGG AGG GG; k-RAS: AGG GCG GTG TGG GAA GAG GGA AGA GGG GGA GG; h-TELO: AGG GTT AGG GTT AGG GTT AGG GT; BCL-2: GGG CGC GGG AGG AAG GGG GCG GG; dsDNA: TAT AGC TAT A Heg TA TAG CTA. The sequences were used as received without further purification.

The oligonucleotide solutions were annealed in pH 7.4 PBS (phosphate-buffered saline) that contained KH_2_PO_4_/K_2_HPO_4_ (10 mM) and potassium fluoride (KF, 100 mM). The oligonucleotide solutions were placed in a dry-block heater at 95 °C for exactly 5 min, before being allowed to cool to room temperature and stored at 4 °C for at least 24 h prior to use. The samples were annealed to 10 μM, then diluted to desired concentration (3–10 μM range) and used without further purification. The exact concentration of each oligonucleotide after annealing (and the corresponding diluted batches) was determined by measuring the absorbance for the peak corresponding to λ_max_. The extinction coefficients were provided by the manufacturer. The following values were used (given in L mol^−1^ cm^−1^): c-KIT2: 199,100; c-KIT1: 213,000; k-RAS: 341,000; BCL-2: 221,000; h-TELO: 237,000; dsDNA: 6600.

The concentration of the DNA sequence used in each experiment is given in the Supplementary Materials.

Ultrapure water from a Milli-Q^®^ water system was used in all CD experiments. All other reagents and solvents were supplied by either Sigma-Aldrich (Gillingham, UK), VWR (Lutterworth, UK) or TCI (Oxford, UK) and used as received. Microwave-assisted reactions were conducted in a CEM^®^ microwave reactor. Samples for NMR analyses were prepared using dimethylsulfoxide (DMSO-*d*_6_). The ^1^H and ^13^C NMR spectra were acquired at 500 and 126 MHz, respectively, using an Agilent ProPulse spectrometer (Santa Clara, CA, USA). The data was recorded at 298 K, and the spectra were referenced to the residual solvent peak. Coupling constants are reported in Hertz (Hz), and signal multiplicity is denoted as singlet (s), broad singlet (br s), doublet (d), doublet of doublets (dd), triplet (t), quartet (q), multiplet (m). Chemical shifts are reported in parts per million (ppm). The nanospray ionization (NSI) spectra (negative or positive ion, as specified) were recorded on an LTQ Orbitrap XL hybrid FTMS instrument. The synthetic route for all NDIs followed a previously reported procedure [35].

The synthesis and characterization data for l-NDIs is reported in our previous work [35]. ^1^H and ^13^C NMR and MS spectra of both d- and l-NDIs are provided for completeness in Supplementary Material Section 1 (pages S2–S14).

### 3.1. Synthesis of N_α_-Boc-d-Lys and N_ε_-Boc-d-Lys NDIs

An 8-mL microwave tube was charged with 1,4,5,8-naphthalenetetracarboxylic dianhydride (NDA) and dissolved in dry DMF (5 mL). The corresponding amino acid was added to the formed suspension along with dry Et_3_N (0.2 mL). The mixture was sonicated until it became a homogeneous solution. The reaction mixture was heated under microwave irradiation for 5 min at 140 °C. The solvent was removed under reduced pressure to yield a brown residue, which was further suspended in minimum amount of acetone. The suspension was added dropwise to a vigorously stirred solution of 1 M HCl_(aq.)_ (200 mL). The resulting precipitate was filtered off and dried in vacuum to yield a light brown colored solid.

***N*_α_-Boc-d-Lys NDI:** The reaction was performed on 1 equivalent of NDA (200 mg, 0.746 mmol) and 2.1 equivalents of *N*_α_-Boc-d-lysine (386 mg, 1.567 mmol) by using the general procedure. Yield: 524 mg, 92%; **^1^H NMR (500 MHz, DMSO-*d*_6_)**: δ 12.40 (br s, 2H), 8.67 (s, 4H), 7.03 (d, *J*_(H,H)_ = 8.1 Hz, 2H), 4.05 (t, *J*_(H,H)_ = 7.4 Hz, 4H), 3.88–3.79 (m, 2H), 1.76–1.50 (m, 8H), 1.36–1.30 (m, 22H); **^13^C NMR (126 MHz, DMSO-*d*_6_)**: δ 174.6, 163.0, 156.0, 130.8, 126.6, 126.5, 78.3, 53.7, 36.2, 31.9, 28.6, 27.5, 23.6; **FTMS-NSI**: *m*/*z* calcd for C_36_H_44_N_4_O_12_: 723.2883 [C_36_H_44_N_4_O_12_-H]^−^, found: 723.2873. 

***N***_ε_**-Boc-d-Lys NDI:** The reaction was performed on 1 equivalent of NDA (200 mg, 0.746 mmol) and 2.1 equivalents of *N*_ε_-Boc-d-lysine (386 mg, 1.567 mmol) by using the general procedure. Yield: 554 mg, 98%; **^1^H NMR (500 MHz, DMSO-*d*_6_)**: δ 12.85 (br s, 2H), 8.75 (s, 4H), 6.67 (t, *J*_(H,H)_ = 6.0 Hz, 2H), 5.53 (dd, *J*_(H,H)_ = 9.4, 5.0 Hz, 2H), 2.85–2.79 (m, 4H), 2.27–2.17 (m, 4H), 2.10–2.01 (m, 4H), 1.21 (s, 22H); **^13^C NMR (126 MHz, DMSO-*d*_6_)**: δ 171.1, 162.8, 155.9, 131.7, 126.8, 126.4, 77.6, 54.0, 36.3, 31.2, 29.8, 28.6, 23.7; **FTMS-NSI**: *m*/*z* calcd for C_36_H_44_N_4_O_12_: 723.2883 [C_36_H_44_N_4_O_12_-H]^−^, found: 723.2874.

### 3.2. Synthesis of N_α_-d-Lys and N_ε_-d-Lys NDIs

The deprotected derivatives (*N*_α_-d-Lys and *N*_ε_-d-Lys NDIs) were obtained from *N*_α_-Boc-d-Lys and *N*_ε_-Boc-d-Lys NDIs via the following general procedure: a 25-mL round-bottomed flask equipped with a stirrer bar was charged with starting material (NDI) suspended in CH_2_Cl_2_ (5 mL) and trifluoroacetic acid (5 mL) was added to the suspension. The reaction mixture was stirred at room temperature for 3 h. The solvents were removed under reduced pressure, and the residue was subsequently washed with diethyl ether (2 × 30 mL), filtered, and vacuum dried to yield a light brown solid in both cases.

***N*_α_-d-Lys NDI:** The aforementioned general procedure was followed using *N*_α_-Boc-d-Lys NDI (394 mg). Yield: 331 mg, 85%; **^1^H NMR (500 MHz, DMSO-*d*_6_)**: δ 13.05 (br s, 2H), 8.66 (s, 4H), 8.16 (br s, 6H), 4.04 (t, *J*_(H,H)_ = 7.6 Hz, 4H), 3.92–3.84 (m, 2H), 1.88–1.72 (m, 8H), 1.72–1.60 (m, 4H); **^13^C NMR (126 MHz, DMSO-*d*_6_)**: δ 171.5, 163.1, 158.9, 158.4, 130.9, 126.8, 126.6, 52.3, 50.2, 30.2, 27.4, 24.2, 22.4; **FTMS-NSI**: *m*/*z* calcd for C_26_H_29_N_4_O_8_: 525.1993 [C_26_H_29_N_4_O_8_-H]^+^, found: 525.1980.

***N*_ε_-d-Lys NDI**: The aforementioned general procedure was following using *N*_ε_-Boc-d-Lys NDI (275 mg). Yield: 176 mg, 62%; **^1^H NMR (500 MHz, DMSO-*d*_6_)**: δ 12.39 (br s, 2H), 8.74 (s, 4H), 7.59 (br s, 6H), 5.53 (dd, *J*_(H,H)_ = 8.9, 5.3 Hz, 2H), 3.37 (t, *J*_(H,H)_ = 7.0 Hz, 4H), 2.31–2.23 (m, 4H), 2.08–2.00 (m, 4H), 1.08 (q, *J*_(H,H)_ = 7.0 Hz, 4H); **^13^C NMR (126 MHz, DMSO-*d*_6_)**: δ 171.1, 162.9, 131.7, 126.9, 126.5, 53.8, 39.1, 36.2, 28.5, 27.3, 23.4. The MS data could not be acquired because of the aggregation issue. 

### 3.3. Variable-Temperature CD Studies

CD and UV-vis experiments were performed on an Applied Photophysics Chirascan Circular Dichroism Spectrophotometer (Leatherhead, UK) equipped with a Peltier temperature controller. The following parameters were used for full spectra measurements: wavelength scanning range 220–420 nm; temperature 5.0 °C; scanning increments 1 nm; monochromator bandwidth 2.5 nm; sampling time-per-point 0.5 s; pathlength cuvette 1 mm.

In all variable-temperature (VT) studies, the ligand (10 equivalents) was added to the DNA sequence solution previously annealed. Any samples with a maximum ellipticity <4.0 mdeg were transferred to a quartz cuvette with a longer pathlength (2, 5 and 10 mm cuvettes were used). For VT experiments, a single-wavelength spectrum was recorded using the wavelength with the maximum ellipticity (λ_max_) with the following parameters: temperature range 5–95 °C with 1 °C increments; temperature slope 1 °C min^−1^; temperature equilibration time 45 s; monochromator bandwidth 2.5 nm; sampling time-per-point 2.0 s. The VT CD data was processed using QtiPlot^®^ (version 0.9.8.9, București, Romania), and fitted to the Boltzmann equation (the spectra are given in the Supplementary Materials).

## 4. Conclusions

**Figure 8:**
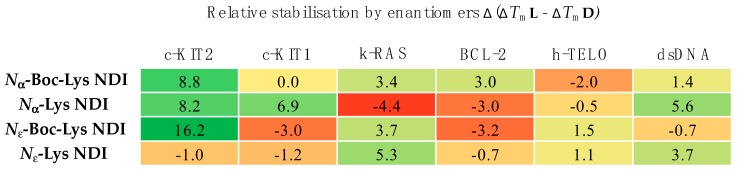
Heat map of the differential stabilization of the studied DNA sequences by the NDI enantiomers. The values represent the difference between the Δ*T*_m_ for the l-enantiomer and the Δ*T*_m_ for the d-enantiomers with each oligonucleotide. Different concentrations were used for N_ε_-Lys NDIs (150 μM for l-NDI and 75 μM for d-NDI); DNA concentration was kept similar for all measurements. All the values are in °C as determined from VT CD studies.

We have synthesized the d-enantiomers of l-Lys NDIs (with chiral center proximal and distal from the polyaromatic unit) and screened them against G4-forming sequences in telomeres (h-TELO) and the oncogene promoters c-KIT1, c-KIT2, k-RAS and BCL-2. To the best of our knowledge, this is the first study to explore the effect of point chirality in metal-free ligands and chiral recognition toward stabilizing G4 DNA sequences. The heat map table in Figure 8 summarizes the potential of l-NDIs (a) vs. d-NDIs (b) as G4 and dsDNA binders.

Enantioselectivity was observed for certain ligand/quadruplex pairs adopting parallel or hybrid conformations. The biggest stabilization difference between the enantiomers was observed for *N*_ε_-Boc-Lys NDI interacting with c-KIT2, where the l-enantiomer stabilizes the quadruplex sequence 16 °C more than the d-enantiomer; the latter has a mild destabilizing effect on the c-KIT2 oligonucleotide (Table 1). The protected and deprotected *N*_α_-Lys NDI enantiomers showed differential stabilization toward this quadruplex in favor of the l-enantiomer. All of the NDI ligands displayed enantiomeric differentiation in the interaction with c-KIT1, k-RAS and BCL-2 with the exception of *N*_α_-Boc-Lys NDI and c-KIT1, and *N*_ε_-Lys NDI with both c-KIT1 and BCL-2. h-TELO does not have a strong interaction with any of the NDI ligands studied.

The enantioselectivity observed cannot be explained by the aromatic π-π stacking of the NDI core on the terminal G-tetrads, thus implying that the sidechain interaction with the loops is the cause of this selectivity. This study paves the way toward further exploration of chiral ligands for G4 DNA with the hope of developing a new generation of G-quadruplex sequence selective anticancer drugs.

## Figures and Tables

**Table 1:** The *T*_m_ values of c-KIT2 only compared to the *T*_m_ of (**a**) c-KIT2-Boc-protected-NDIs and (**b**) c-KIT2-deprotected-NDIs as determined from VT CD studies.

(a)		(b)	
Sequence/Assembly	*T*_m_ (°C)	Sequence/Assembly	*T*_m_ (°C)
c-KIT2 only	78.2 ± 2.7 ^a^/78.9 ± 0.8 ^b^	c-KIT2 only	78.2 ± 2.7 ^a^/78.9 ± 0.8 ^b^
*N*_ε_-Boc-L-Lys NDI	92.8 ± 5.9	*N*_ε_-L-Lys NDI	75.1 ± 0.9
*N*_ε_-Boc-D-Lys NDI	77.3 ± 0.4	*N*_ε_-D-Lys NDI	76.8 ± 0.3
*N*_α_-Boc-L-Lys NDI	88.4 ± 8.4	*N*_α_-L-Lys NDI	88.7 ± 7.1
*N*_α_-Boc-D-Lys NDI	80.3 ± 0.1	*N*_α_-D-Lys NDI	81.2 ± 1.0

^a^ This value was used for calculating the *T*_m_ for all c-KIT2-L-NDIs assemblies; ^b^ This value was used for calculating the *T*_m_ for all c-KIT2-D-NDIs assemblies.

**Table 2:** The *T*_m_ values of c-KIT1 only compared to the *T*_m_ of (**a**) c-KIT1-Boc-protected-NDIs and (**b**) c-KIT1-deprotected-NDIs as determined from VT CD studies.

(a)		(b)	
Sequence/Assembly	*T*_m_ (°C)	Sequence/Assembly	*T*_m_ (°C)
c-KIT1 only	65.8 ± 0.4 ^a^/65.4 ± 0.4 ^b^	c-KIT1 only	65.8 ± 0.4 ^a^/65.4 ± 0.4 ^b^
*N*_ε_-Boc-L-Lys NDI	64.3 ± 0.4	*N*_ε_-L-Lys NDI	65.6 ± 0.7
*N*_ε_-Boc-D-Lys NDI	66.9 ± 0.2	*N*_ε_-D-Lys NDI	66.4 ± 0.2
*N*_α_-Boc-L-Lys NDI	67.5 ± 1.0	*N*_α_-L-Lys NDI	68.0 ± 0.8
*N*_α_-Boc-D-Lys NDI	67.1 ± 0.2	*N*_α_-D-Lys NDI	69.3 ± 0.3

^a^ This value was used for calculating the *T*_m_ for all c-KIT1-L-NDIs assemblies; ^b^ This value was used for calculating the *T*_m_ for all c-KIT1-D-NDIs assemblies.

**Table 3:** The *T*_m_ values of k-RAS only compared to the *T*_m_ of (**a**) k-RAS-Boc-protected-NDIs and (**b**) k-RAS-deprotected-NDIs as determined from VT CD studies.

(a)		(b)	
Sequence/Assembly	*T*_m_ (°C)	Sequence/Assembly	*T*_m_ (°C)
k-RAS only	58.3 ± 0.6 ^a^/55.8 ± 0.1 ^b^	k-RAS only	58.3 ± 0.6 ^a^/55.8 ± 0.1 ^b^
*N*_ε_-Boc-L-Lys NDI	60.0 ± 0.5	*N*_ε_-L-Lys NDI	62.4 ± 0.4
*N*_ε_-Boc-D-Lys NDI	53.8 ± 0.1	*N*_ε_-D-Lys NDI	54.6 ± 0.1
*N*_α_-Boc-L-Lys NDI	60.6 ± 0.3	*N*_α_-L-Lys NDI	58.7 ± 1.1
*N*_α_-Boc-D-Lys NDI	54.7 ± 0.1	*N*_α_-D-Lys NDI	60.6 ± 0.1

^a^ This value was used for calculating the *T*_m_ for all k-RAS-L-NDIs assemblies; ^b^ This value was used for calculating the *T*_m_ for all k-RAS-D-NDIs assemblies.

**Table 4:** The *T*_m_ values of BCL-2 only compared to the *T*_m_ of (**a**) BCL-2-Boc-protected-NDIs and (**b**) BCL-2-deprotected-NDIs as determined from VT CD studies.

(a)		(b)	
Sequence/Assembly	*T*_m_ (°C)	Sequence/Assembly	*T*_m_ (°C)
BCL-2 only	66.0 ± 0.4 ^a^/66.1 ± 0.4 ^b^	BCL-2 only	66.0 ± 0.4 ^a^/66.1 ± 0.4 ^b^
*N*_ε_-Boc-L-Lys NDI	65.2 ± 0.5	*N*_ε_-L-Lys NDI	67.7 ± 0.4
*N*_ε_-Boc-D-Lys NDI	68.5 ± 0.4	*N*_ε_-D-Lys NDI	68.5 ± 0.3
*N*_α_-Boc-L-Lys NDI	74.9 ± 0.8	*N*_α_-L-Lys NDI	69.7 ± 1.0
*N*_α_-Boc-D-Lys NDI	72.0 ± 0.3	*N*_α_-D-Lys NDI	72.8 ± 0.3

^a^ This value was used for calculating the *T*_m_ for all BCL-2-L-NDIs assemblies; ^b^ This value was used for calculating the *T*_m_ for all BCL-2-D-NDIs assemblies.

**Table 5:** The *T*_m_ values of h-TELO only compared to the *T*_m_ of (**a**) h-TELO-Boc-protected-NDIs and (**b**) h-TELO-deprotected-NDIs as determined from VT CD studies.

(a)		(b)	
Sequence/Assembly	*T*_m_ (°C)	Sequence/Assembly	*T*_m_ (°C)
h-TELO only	62.5 ± 0.3 ^a^/65.8 ± 0.1 ^b^	h-TELO only	62.5 ± 0.3 ^a^/65.8 ± 0.1 ^b^
*N*_ε_-Boc-L-Lys NDI	61.5 ± 0.6	*N*_ε_-L-Lys NDI	64.5 ± 0.4
*N*_ε_-Boc-D-Lys NDI	63.2 ± 0.1	*N*_ε_-D-Lys NDI	66.7 ± 0.2
*N*_α_-Boc-L-Lys NDI	61.8 ± 0.4	*N*_α_-L-Lys NDI	61.4 ± 1.0
*N*_α_-Boc-D-Lys NDI	67.1 ± 0.1	*N*_α_-D-Lys NDI	65.2 ± 0.1

^a^ This value was used for calculating the *T*_m_ for all h-TELO-L-NDIs assemblies; ^b^ This value was used for calculating the *T*_m_ for all h-TELO-D-NDIs assemblies.

**Table 6:** The *T*_m_ values of dsDNA only compared to the *T*_m_ of (**a**) dsDNA-Boc-protected-NDIs and (**b**) dsDNA-deprotected-NDIs as determined from VT CD studies.

(a)		(b)	
Sequence/Assembly	*T*_m_ (°C)	Sequence/Assembly	*T*_m_ (°C)
dsDNA only	55.1 ± 0.8 ^a^/55.6 ± 0.3 ^b^	dsDNA only	55.1 ± 0.8 ^a^/55.6 ± 0.3 ^b^
*N*_ε_-Boc-L-Lys NDI	52.6 ± 0.8	*N*_ε_-L-Lys NDI	56.9 ± 1.5
*N*_ε_-Boc-D-Lys NDI	53.8 ± 0.2	*N*_ε_-D-Lys NDI	53.7 ± 0.3
*N*_α_-Boc-L-Lys NDI	57.0 ± 1.2	*N*_α_-L-Lys NDI	55.8 ± 0.7
*N*_α_-Boc-D-Lys NDI	56.1 ± 0.6	*N*_α_-D-Lys NDI	50.7 ± 0.3

^a^ This value was used for calculating the *T*_m_ for all dsDNA-L-NDIs assemblies; ^b^ This value was used for calculating the *T*_m_ for all dsDNA-D-NDIs assemblies.

## Supplementary Materials

The Supplementary Materials are available online. CD analysis of all DNA sequences with and without the ligand before and during VT CD studies are given in Figures S1–S11; ^1^H, ^13^C NMR, and MS spectra of both l- and d-NDIs are supplied in Section 1 (pages S2–S14). VT CD melting curves and the corresponding Boltzmann fittings are provided in Section 3, Figures S12–S18. Size exclusion chromatography (SEC) analysis of the all the DNA sequences is presented in Figure S19.
