# Supplementary material for: Importance of Chiral Recognition in Designing Metal-Free Ligands for G-Quadruplex DNA
Source: Molecules. 2019 Apr 15;24(8):1473. doi: 10.3390/molecules24081473 (PMC6514905; doi:10.3390/molecules24081473)
Supplement: Supplementary file 1 [file molecules-24-01473-s001.pdf]

Supplementary Material

Importance of chiral recognition in designing metal-free ligands for G-quadruplex DNA

Dora M. Răsădean [1], Samuel W. O. Harrison [1], Isobel R. Owens [1], Aucéanne Miramont [1], Frances M. Bromley [1] and G. Dan Pantoș [1,*]

[1] Department of Chemistry, University of Bath, Claverton Down, Bath, BA2 7AY, U.K.

* Correspondence: g.d.pantos@bath.ac.uk; Tel.: +44-1225-384-376

Table of Contents

1. Characterization of D-NDIs and L-NDIs S2

2. Conformation analysis S14

3. CD thermal melting studies S24

3.1. c-KIT2 .... S25

3.2. c-KIT1 .... S26

3.3. k-RAS S27

3.4. BCL-2 .... S28

3.4.1. BCL-2 with L-NDIs S28

3.4.2. BCL-2 with D-NDIs S29

3.5. h-TELO S30

3.6. dsDNA S31

4. Size-exclusion chromatography analysis S32

5. References S35

1. Characterization of D-NDIs and L-NDIs

^{1}H and ^{13}C NMR spectra of D-NDIs are provided below in the order in which their synthesis is reported in the main text. The ^{1}H and ^{13}C NMR spectra of L-NDIs are also given for completeness (full characterization of L-NDIs is also listed in our previous work) [1]. The peaks coming from residual solvent (DMSO-*d*6) as well as other solvent impurities (*e.g.* dimethylformamide, water, acetone) are labelled with an “x”. Information about NMR setup for both ^{1}H and ^{13}C NMR spectra are given in the paper in Section 3.

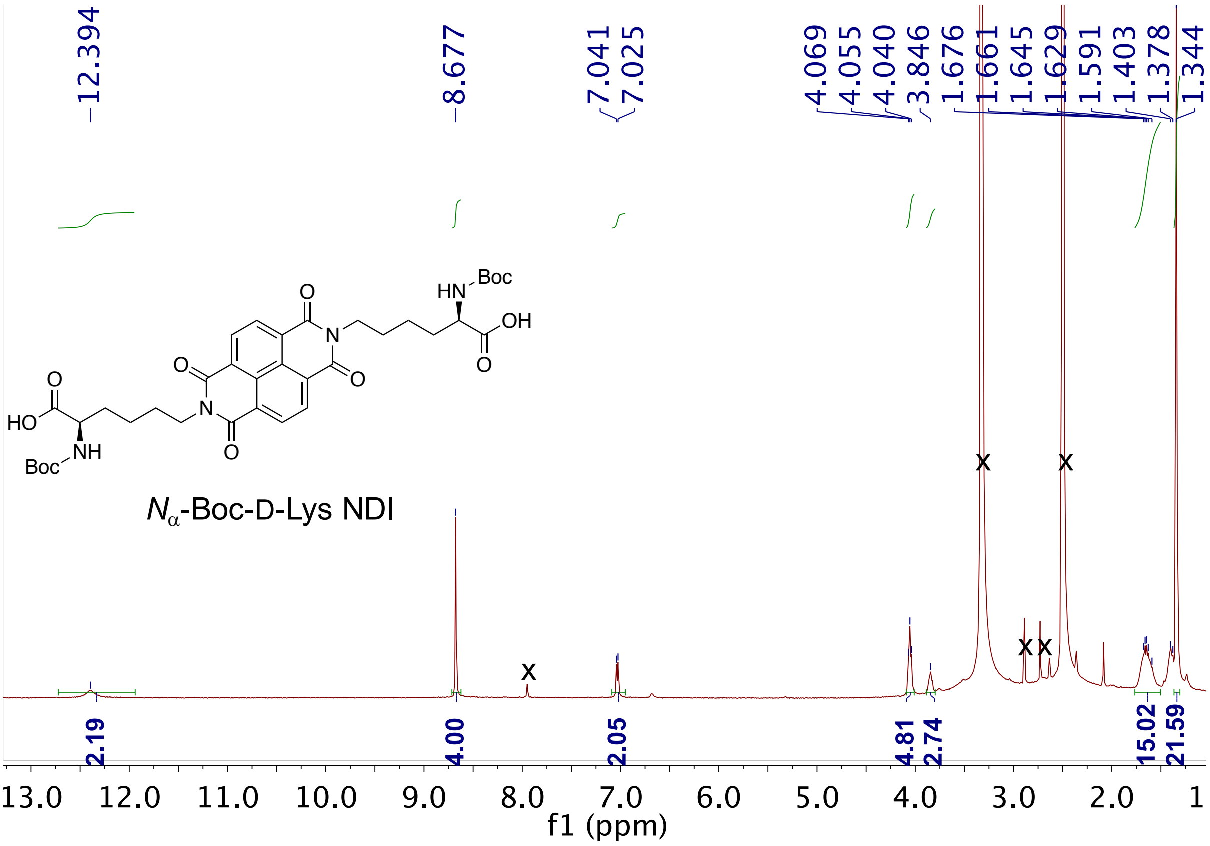

12.394
8.677
7.041
7.025
4.069
4.055
4.040
3.846
1.676
1.661
1.645
1.629
1.591
1.403
1.378
1.344
HN
Boc
OH
O
N
HO
NH
Nα-Boc-D-Lys NDI
x
2.19
4.00
2.05
4.81
2.74
15.02
21.59
13.0 12.0 11.0 10.0 9.0 8.0 7.0 6.0 5.0 4.0 3.0 2.0 1
f1 (ppm)

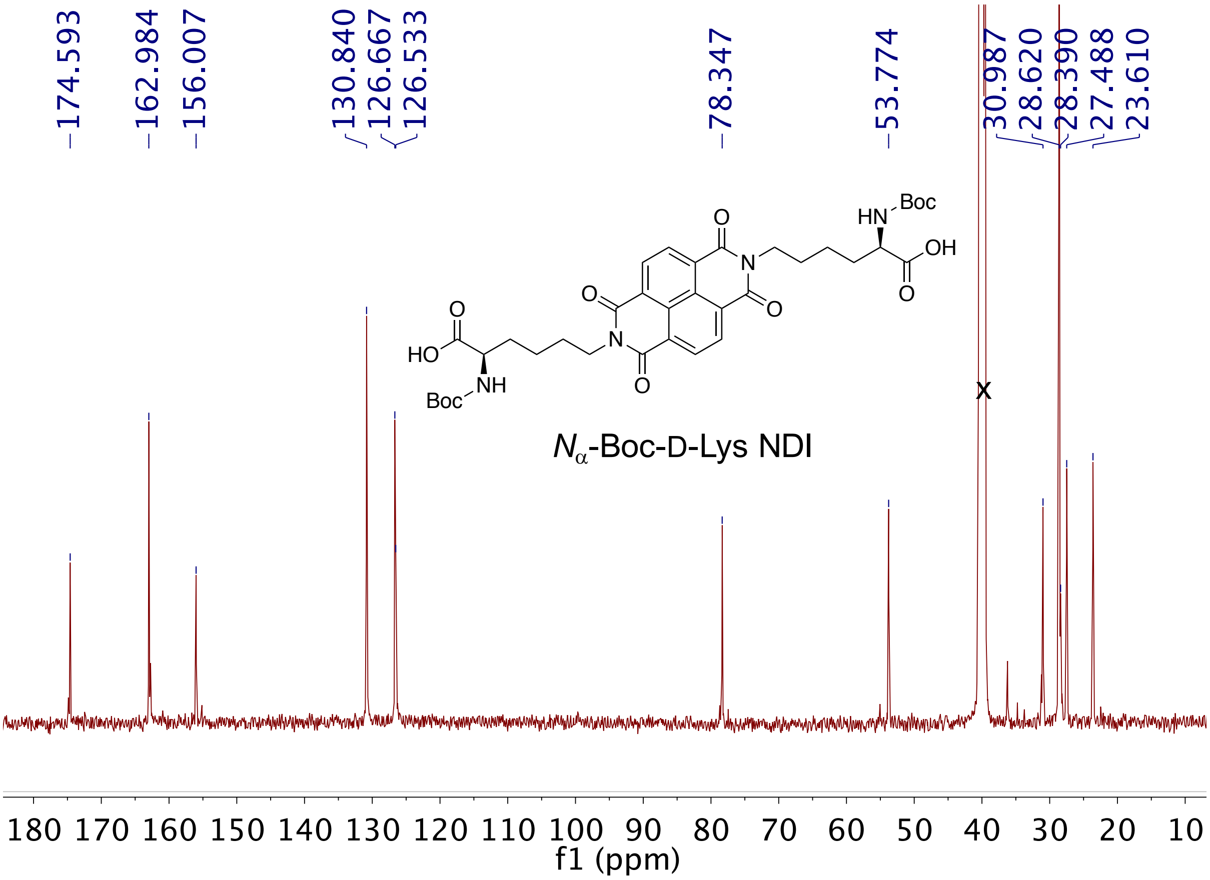

174.593
162.984
156.007
130.840
126.667
126.533
78.347
53.774
30.987
28.620
28.390
27.488
23.610
HN
Boc
OH
O
N
HO
NH
Nα-Boc-D-Lys NDI
x
180 170 160 150 140 130 120 110 100 90 80 70 60 50 40 30 20 10
f1 (ppm)

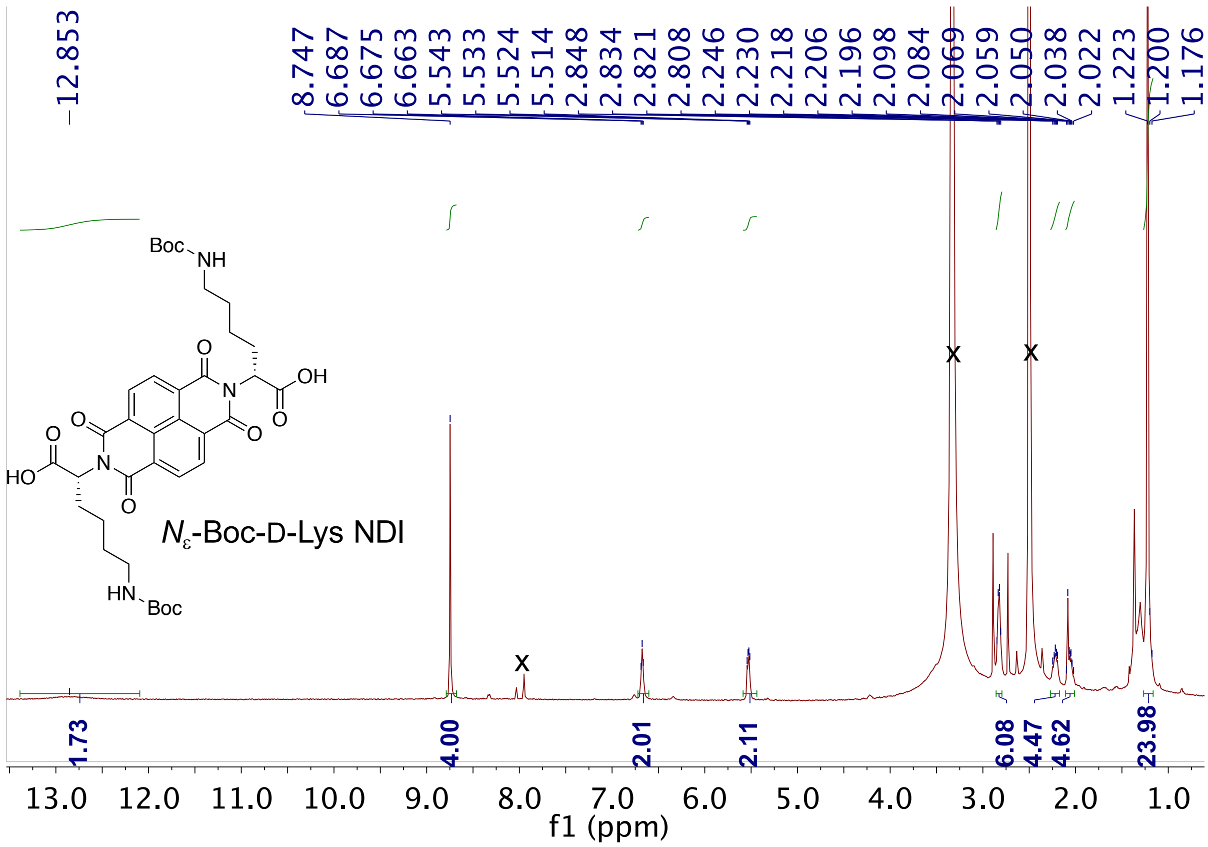
N_ε-Boc-D-Lys NDI
f1 (ppm)

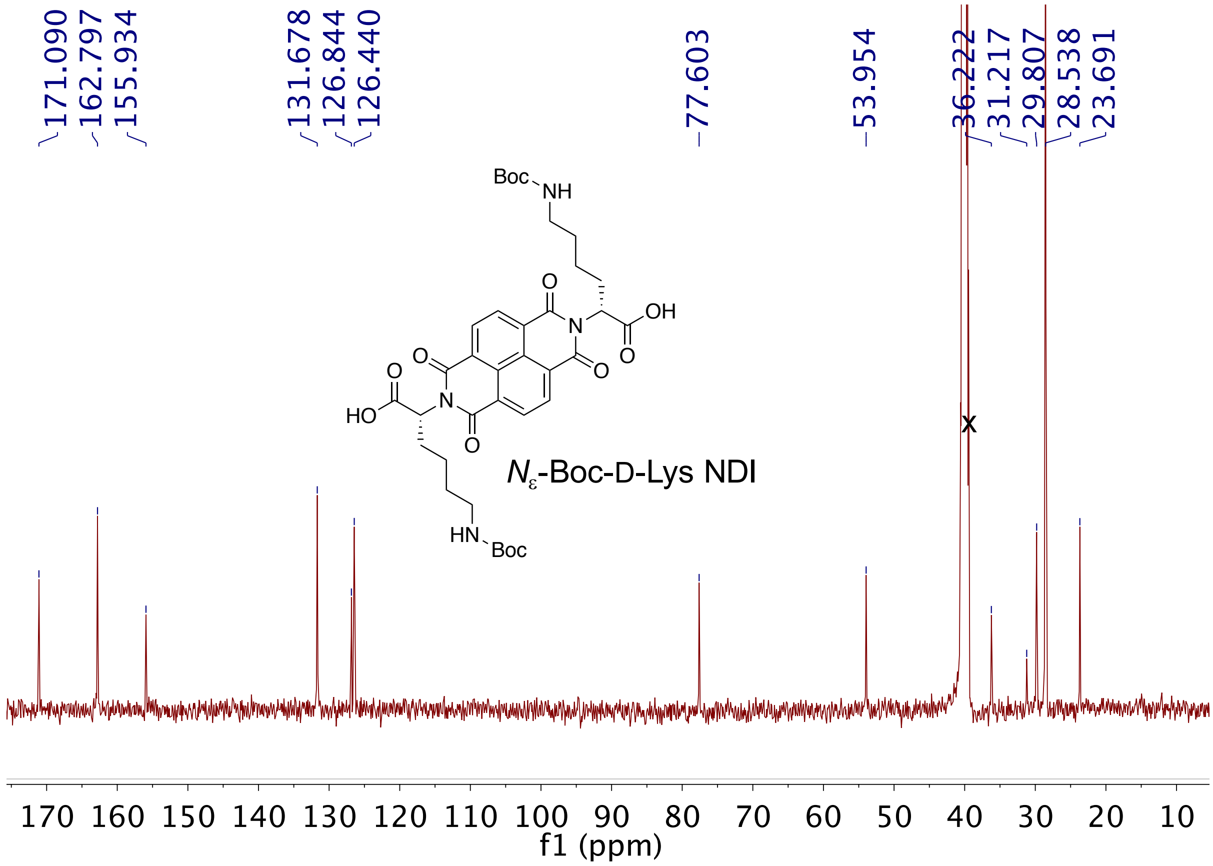
N_ε-Boc-D-Lys NDI
f1 (ppm)

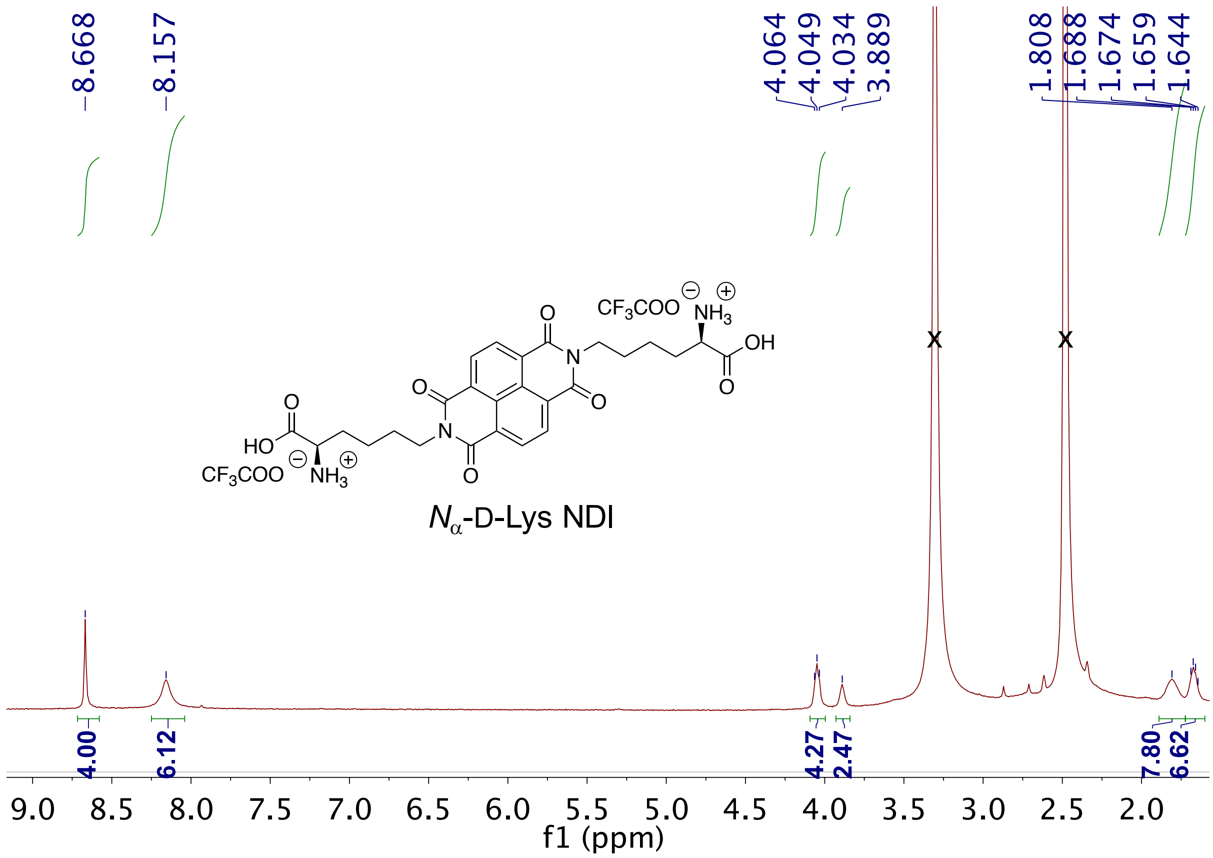
8.668
8.157
4.064
4.049
4.034
3.889
1.808
1.688
1.674
1.659
1.644
CF3COO NH3
OH
HO
N_α-D-Lys NDI
x
x
4.00
6.12
4.27
2.47
7.80
6.62
9.0 8.5 8.0 7.5 7.0 6.5 6.0 5.5 5.0 4.5 4.0 3.5 3.0 2.5 2.0
f1 (ppm)

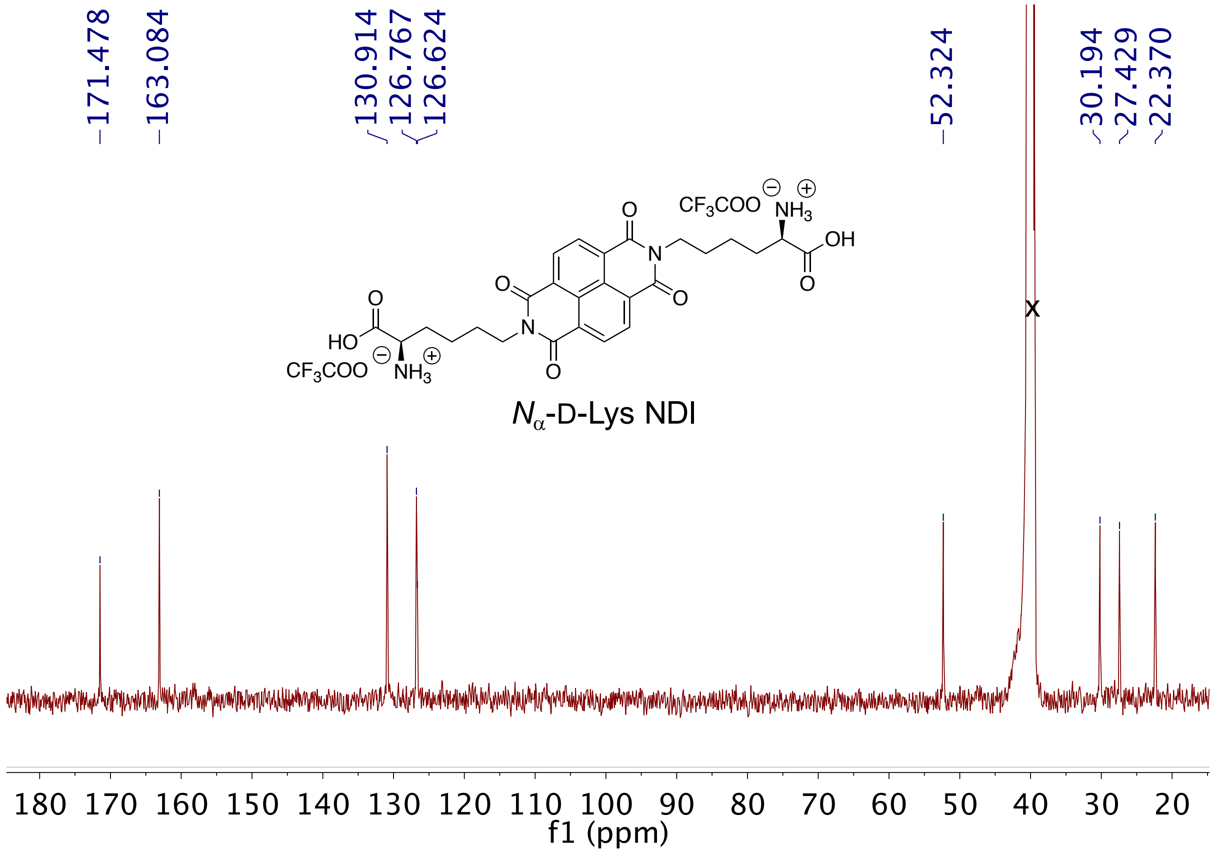
171.478
163.084
130.914
126.767
126.624
52.324
30.194
27.429
22.370
CF3COO NH3
OH
HO
N_α-D-Lys NDI
x
180 170 160 150 140 130 120 110 100 90 80 70 60 50 40 30 20
f1 (ppm)

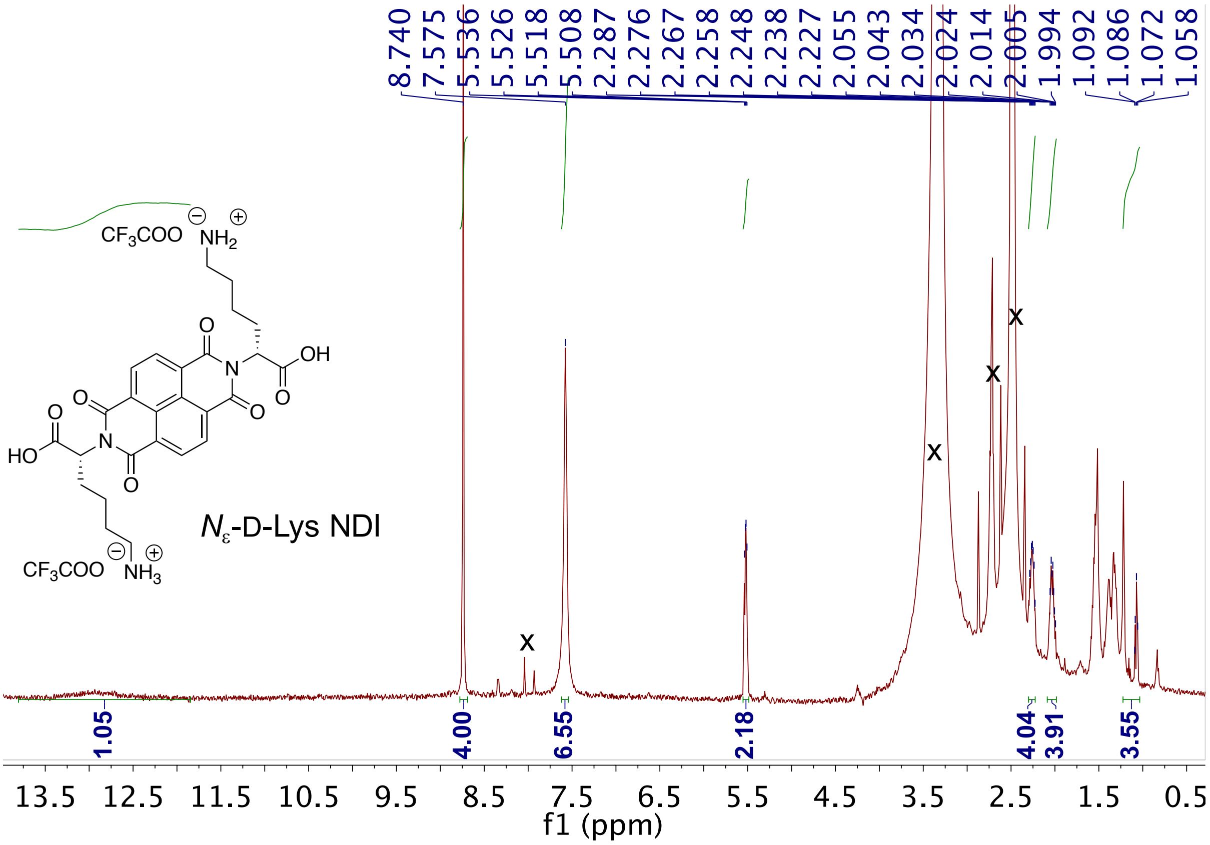
8.740
7.575
5.536
5.526
5.518
5.508
2.287
2.276
2.267
2.258
2.248
2.238
2.227
2.055
2.043
2.034
2.024
2.014
2.005
1.994
1.092
1.086
1.072
1.058
CF3COO NH2
OH
HO
CF3COO NH3
Nε-D-Lys NDI
1.05
4.00
6.55
2.18
4.04
3.91
3.55
13.5 12.5 11.5 10.5 9.5 8.5 7.5 6.5 5.5 4.5 3.5 2.5 1.5 0.5
f1 (ppm)

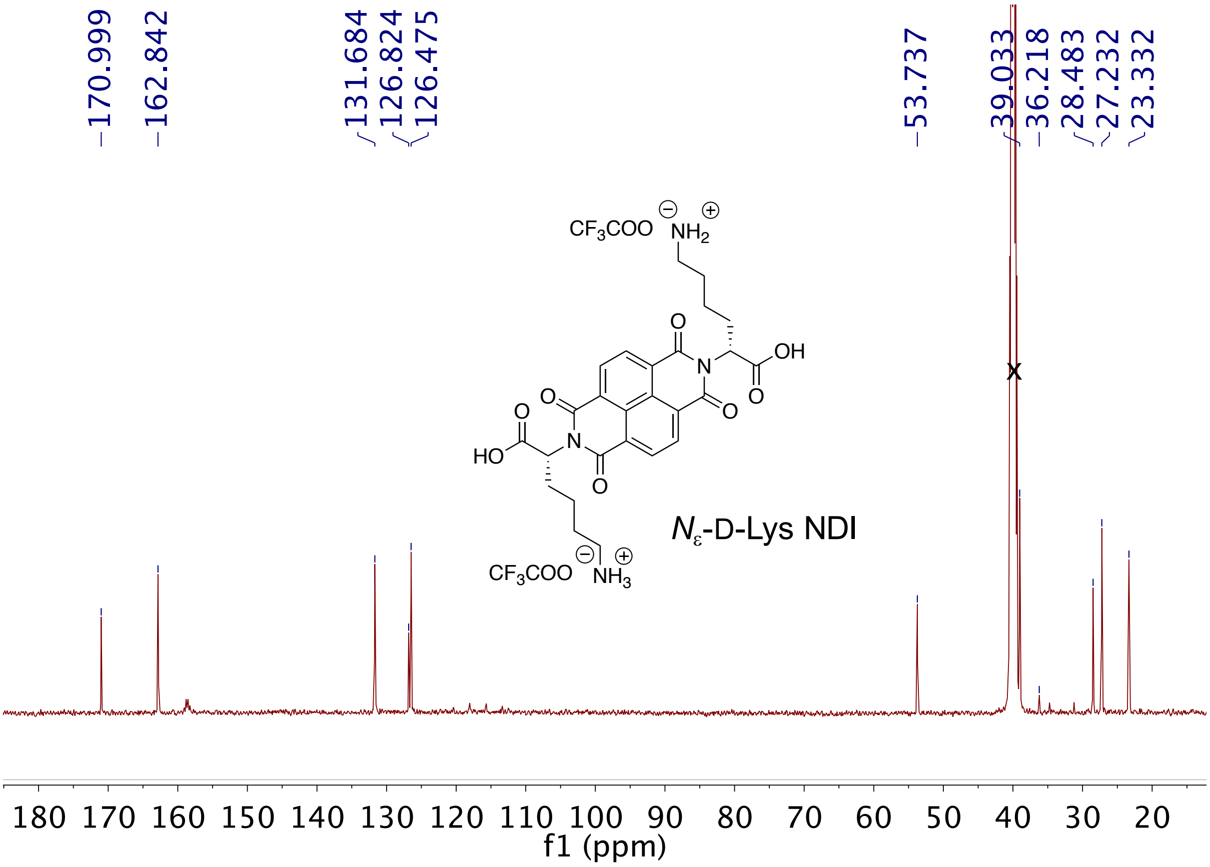
170.999
162.842
131.684
126.824
126.475
53.737
39.033
36.218
28.483
27.232
23.332
CF3COO NH2
OH
HO
CF3COO NH3
Nε-D-Lys NDI
180 170 160 150 140 130 120 110 100 90 80 70 60 50 40 30 20
f1 (ppm)

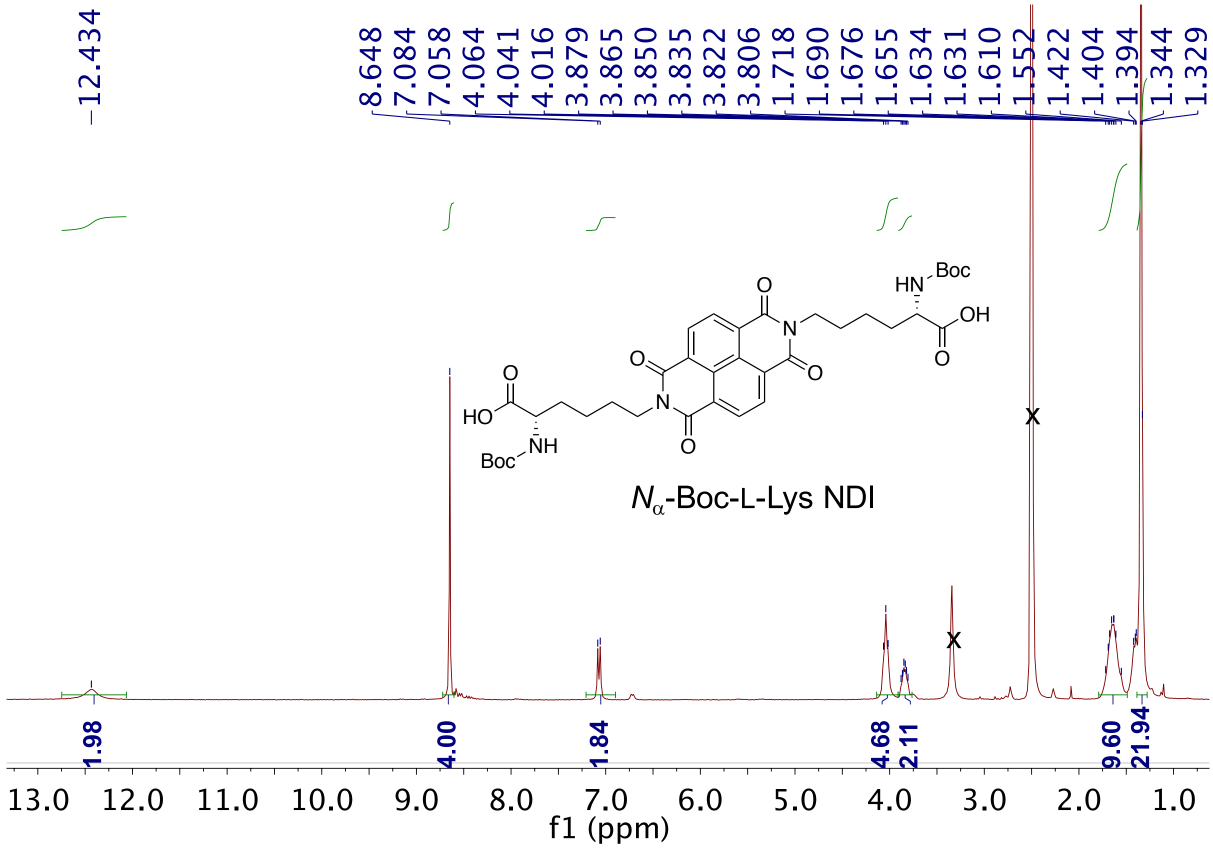
12.434
8.648
7.084
7.058
4.064
4.041
4.016
3.879
3.865
3.850
3.835
3.822
3.806
1.718
1.690
1.676
1.655
1.634
1.631
1.610
1.552
1.422
1.404
1.394
1.344
1.329
Boc
HN
OH
O
N
HO
NH
Boc
N_α-Boc-L-Lys NDI
X
1.98
4.00
1.84
4.68
2.11
9.60
21.94
13.0 12.0 11.0 10.0 9.0 8.0 7.0 6.0 5.0 4.0 3.0 2.0 1.0
f1 (ppm)

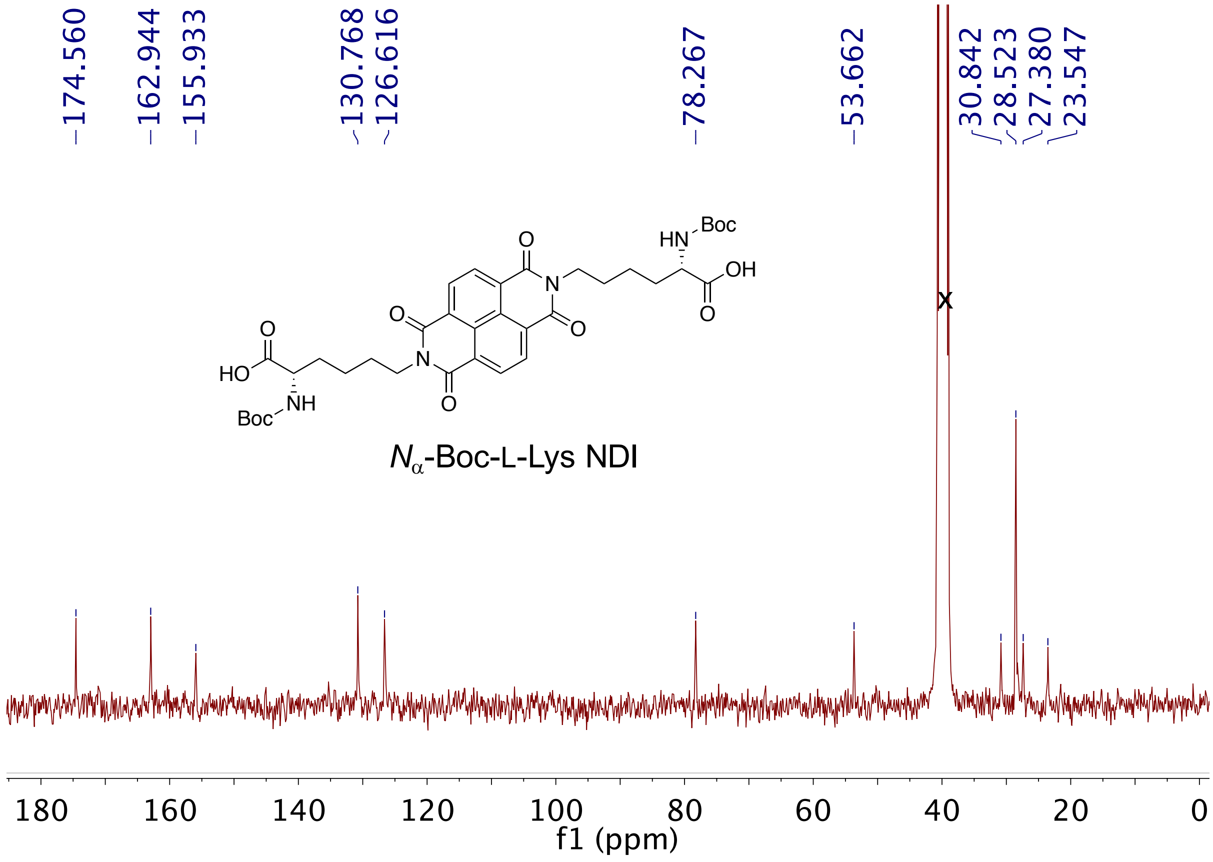
174.560
162.944
155.933
130.768
126.616
78.267
53.662
30.842
28.523
27.380
23.547
Boc
HN
OH
O
N
HO
NH
Boc
N_α-Boc-L-Lys NDI
X
180 160 140 120 100 80 60 40 20 0
f1 (ppm)

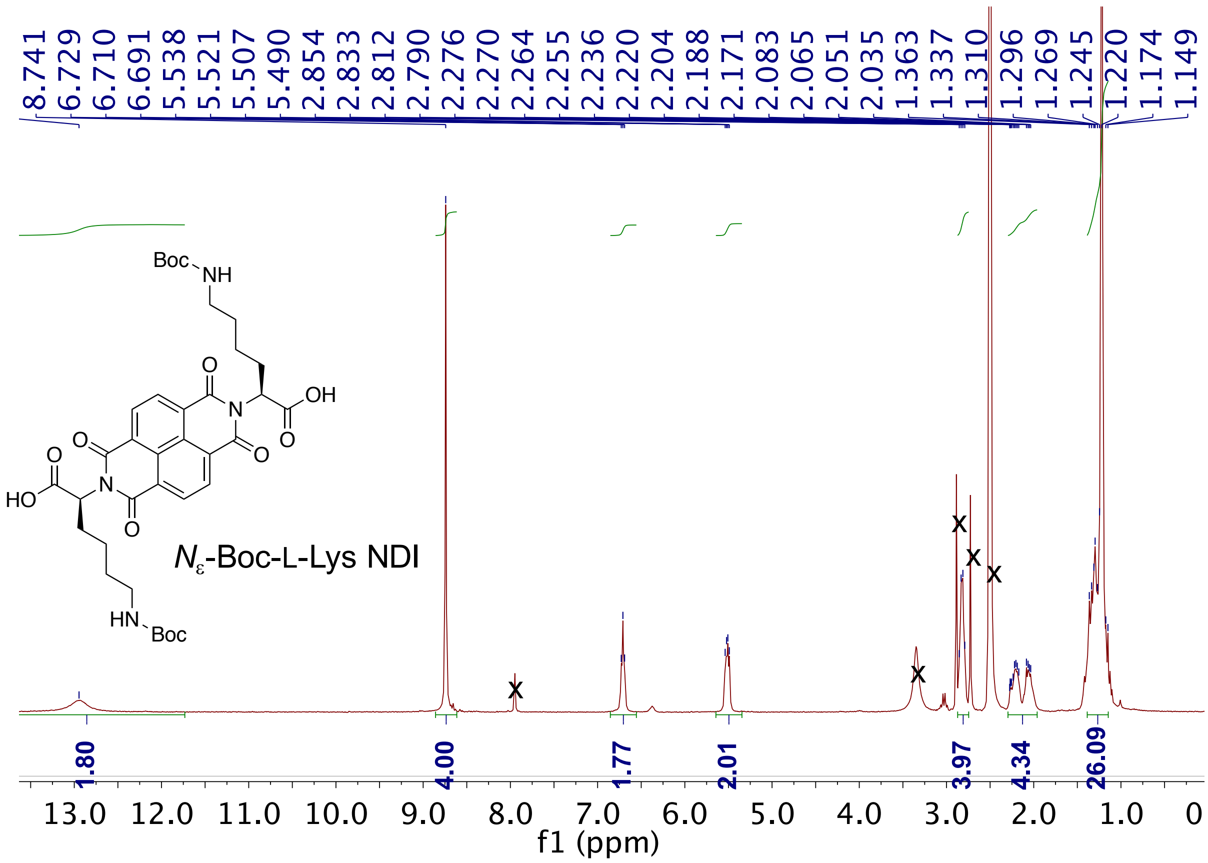
8.741
6.729
6.710
6.691
5.538
5.521
5.507
5.490
2.854
2.833
2.812
2.790
2.276
2.270
2.264
2.255
2.236
2.220
2.204
2.188
2.171
2.083
2.065
2.051
2.035
1.363
1.337
1.310
1.296
1.269
1.245
1.220
1.174
1.149
Boc
NH
O
N
OH
O
O
O
O
N
HO
O
O
HN
Boc
N_ε-Boc-L-Lys NDI
x
x
x
x
x
1.80
4.00
1.77
2.01
3.97
4.34
26.09
13.0
12.0
11.0
10.0
9.0
8.0
7.0
6.0
5.0
4.0
3.0
2.0
1.0
0
f1 (ppm)

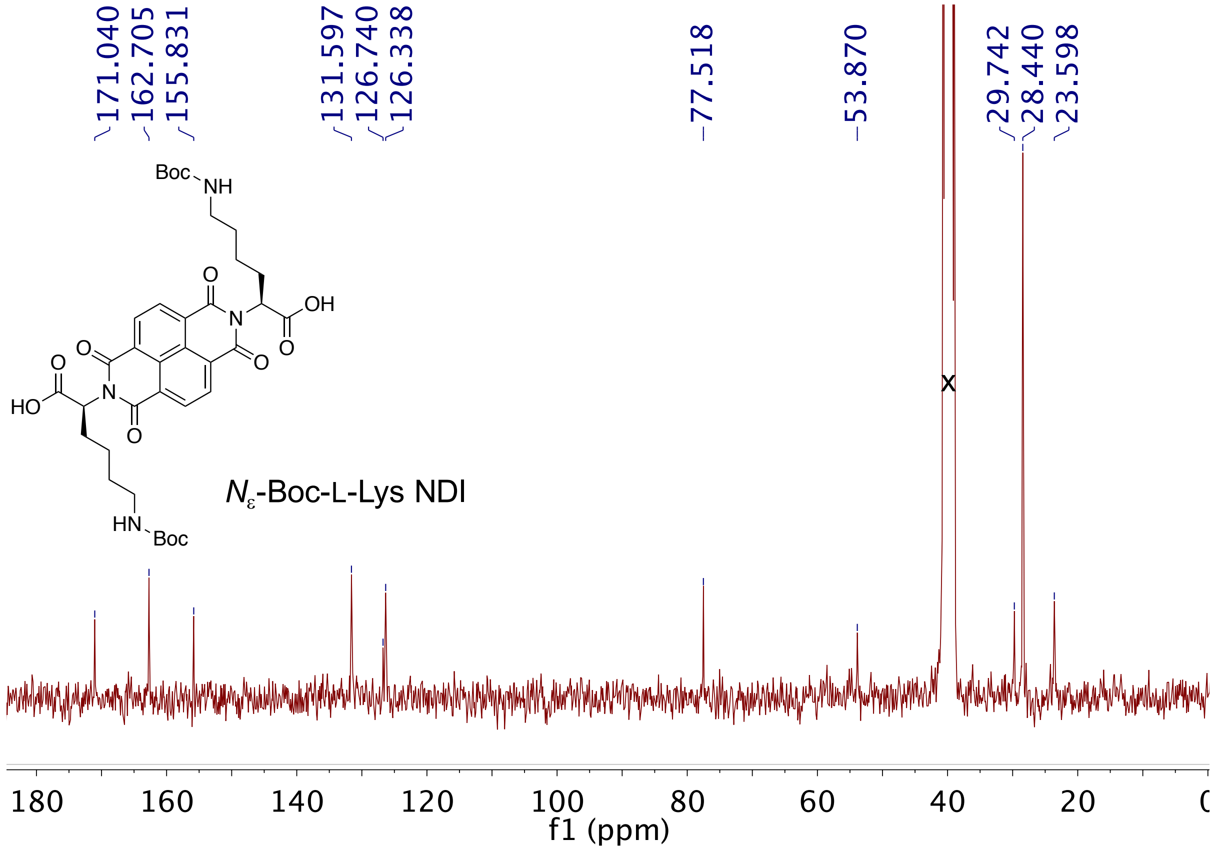
171.040
162.705
155.831
131.597
126.740
126.338
77.518
53.870
29.742
28.440
23.598
Boc
NH
O
N
OH
O
O
O
O
N
HO
O
O
HN
Boc
N_ε-Boc-L-Lys NDI
x
180
160
140
120
100
80
60
40
20
f1 (ppm)

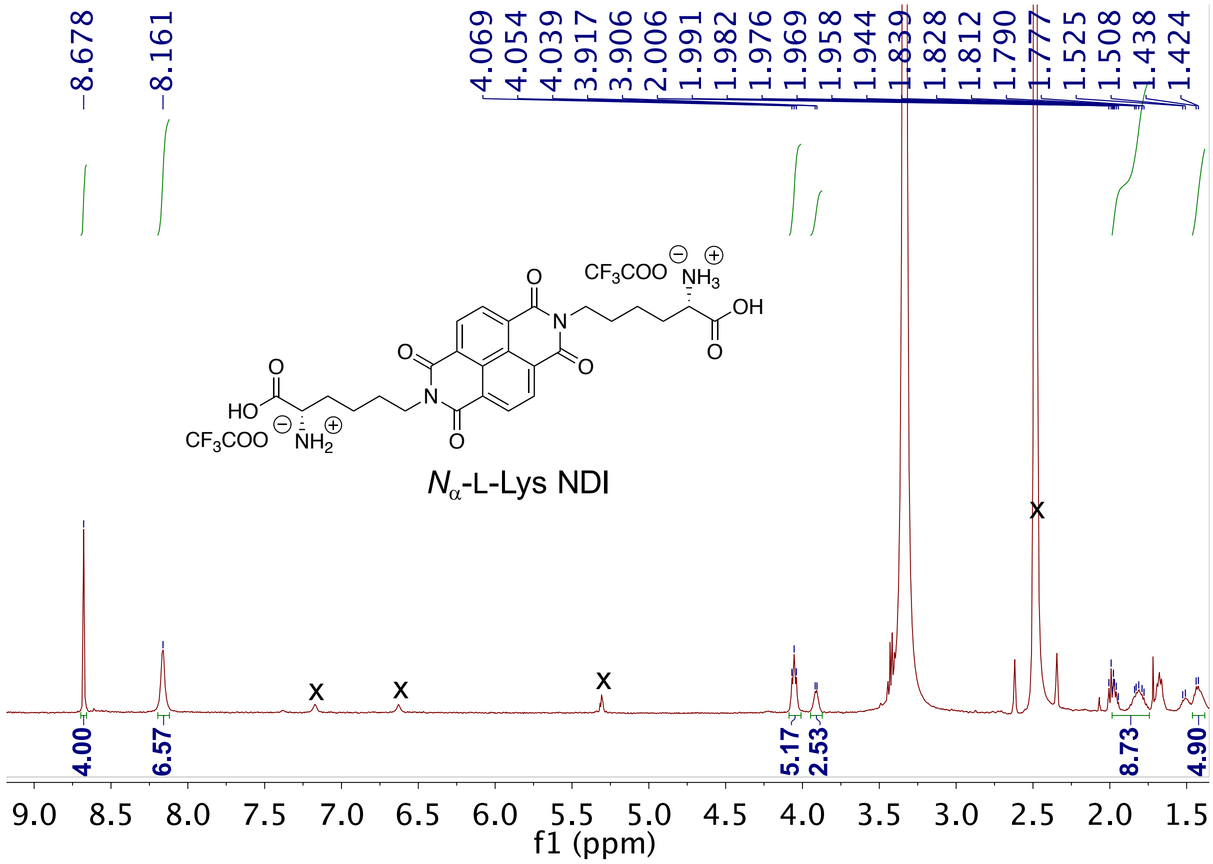
-8.678
-8.161
4.069
4.054
4.039
3.917
3.906
2.006
1.991
1.982
1.976
1.969
1.958
1.944
1.839
1.828
1.812
1.790
1.777
1.525
1.508
1.438
1.424
CF3COO NH3
OH
O
HO
CF3COO NH2
N_α-L-Lys NDI
x
x
x
x
4.00
6.57
5.17
2.53
8.73
4.90
9.0 8.5 8.0 7.5 7.0 6.5 6.0 5.5 5.0 4.5 4.0 3.5 3.0 2.5 2.0 1.5
f1 (ppm)

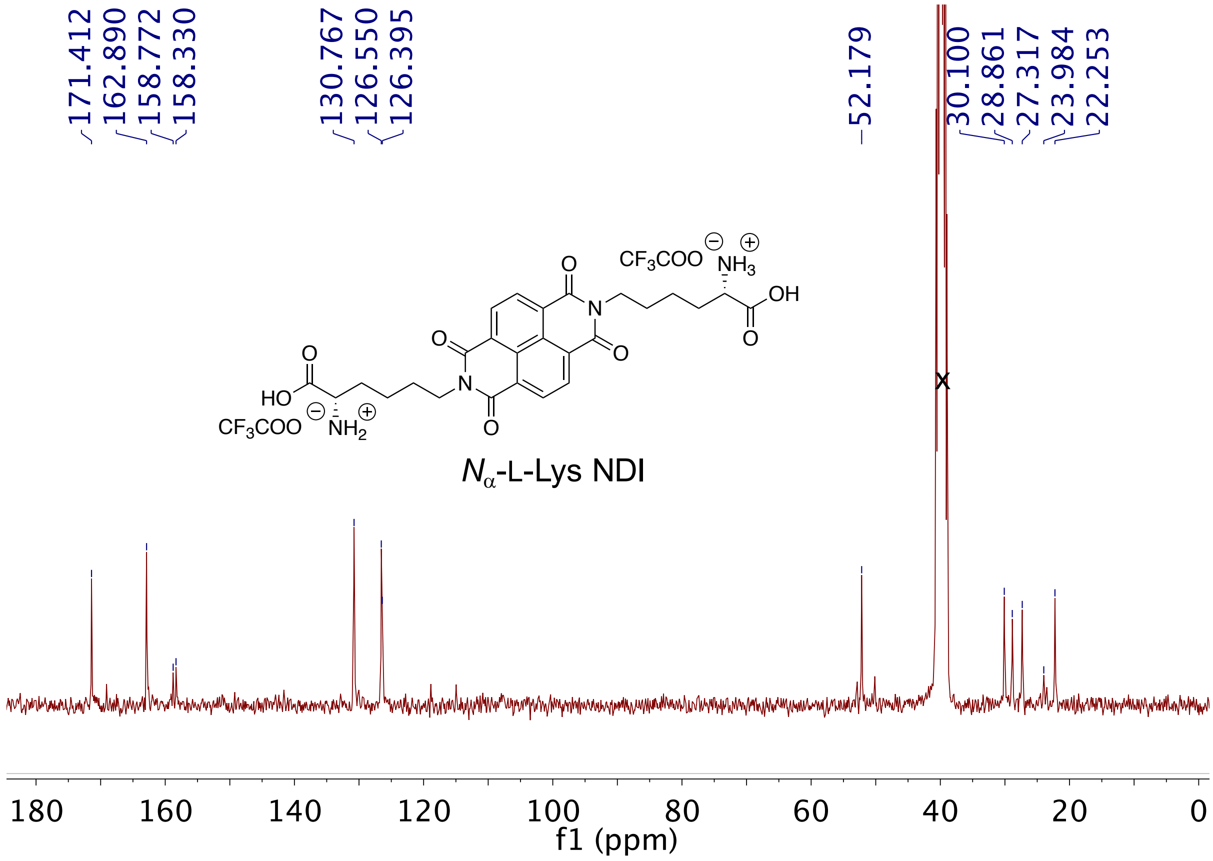
171.412
162.890
158.772
158.330
130.767
126.550
126.395
-52.179
30.100
28.861
27.317
23.984
22.253
CF3COO NH3
OH
O
HO
CF3COO NH2
N_α-L-Lys NDI
X
180 160 140 120 100 80 60 40 20 0
f1 (ppm)

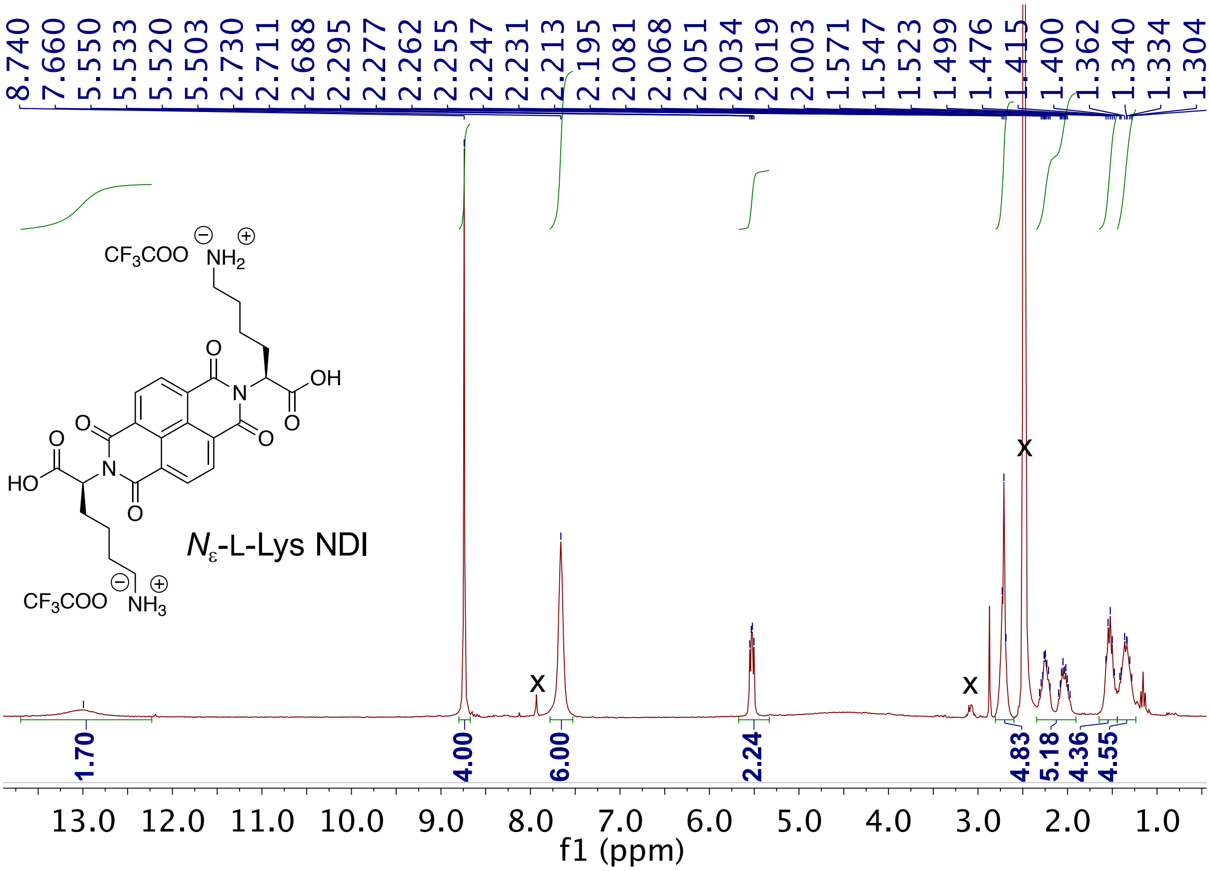
8.740 7.660 5.550 5.533 5.520 5.503 2.730 2.711 2.688 2.295 2.277 2.262 2.255 2.247 2.231 2.213 2.195 2.081 2.068 2.051 2.034 2.019 2.003 1.571 1.547 1.523 1.499 1.476 1.415 1.400 1.362 1.340 1.334 1.304
CF3COO NH2
OH
HO
N
O
Nε-L-Lys NDI
CF3COO NH3
x
1.70 4.00 6.00 2.24 4.83 5.18 4.36 4.55
13.0 12.0 11.0 10.0 9.0 8.0 7.0 6.0 5.0 4.0 3.0 2.0 1.0
f1 (ppm)

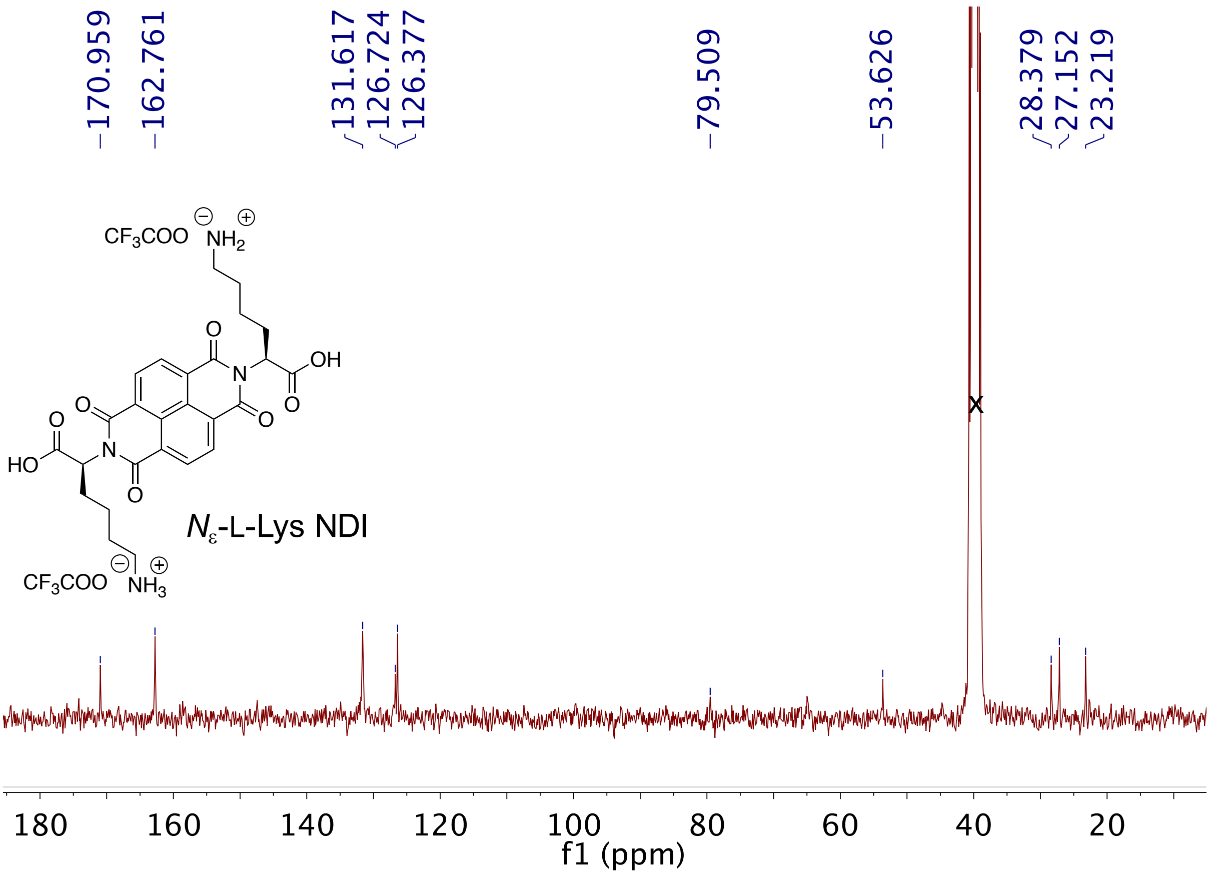
170.959 162.761 131.617 126.724 126.377 79.509 53.626 28.379 27.152 23.219
CF3COO NH2
OH
HO
N
O
Nε-L-Lys NDI
CF3COO NH3
x
180 160 140 120 100 80 60 40 20
f1 (ppm)

MS spectra for both D- and L-NDIs are provided below in the order in which their synthesis is reported in the main text. Experimental details are given in the main text in Section 3.

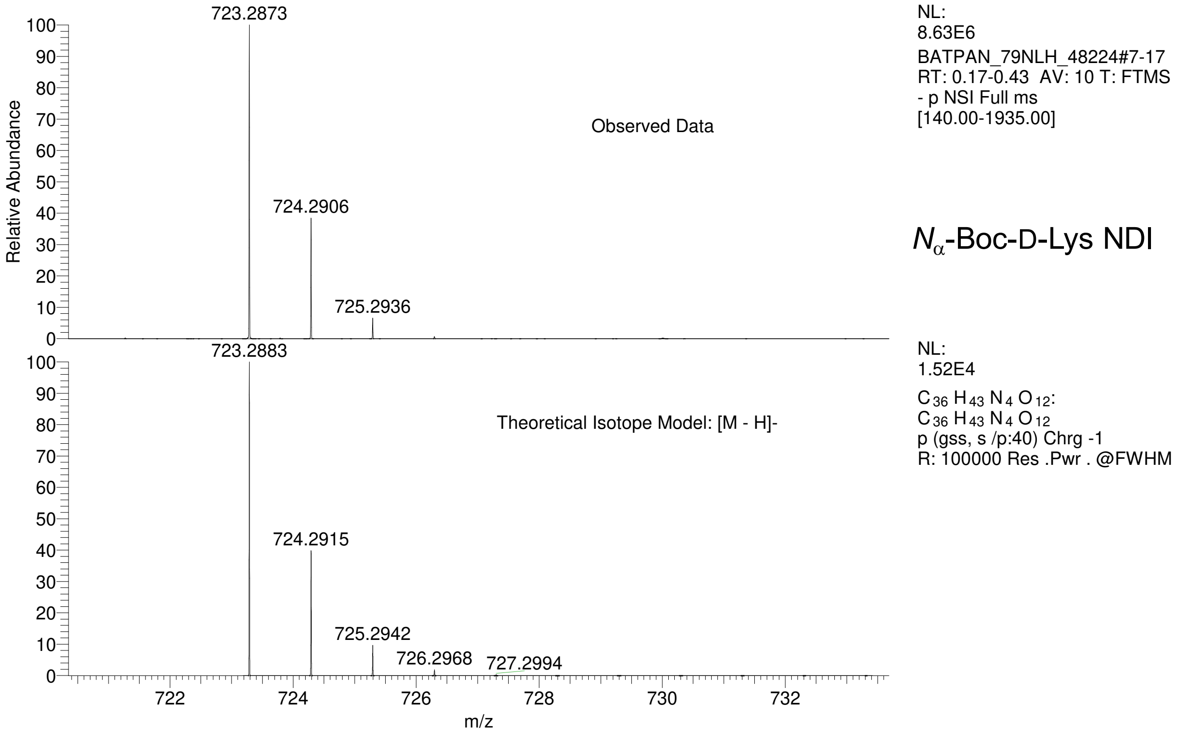

N_α-Boc-D-Lys NDI

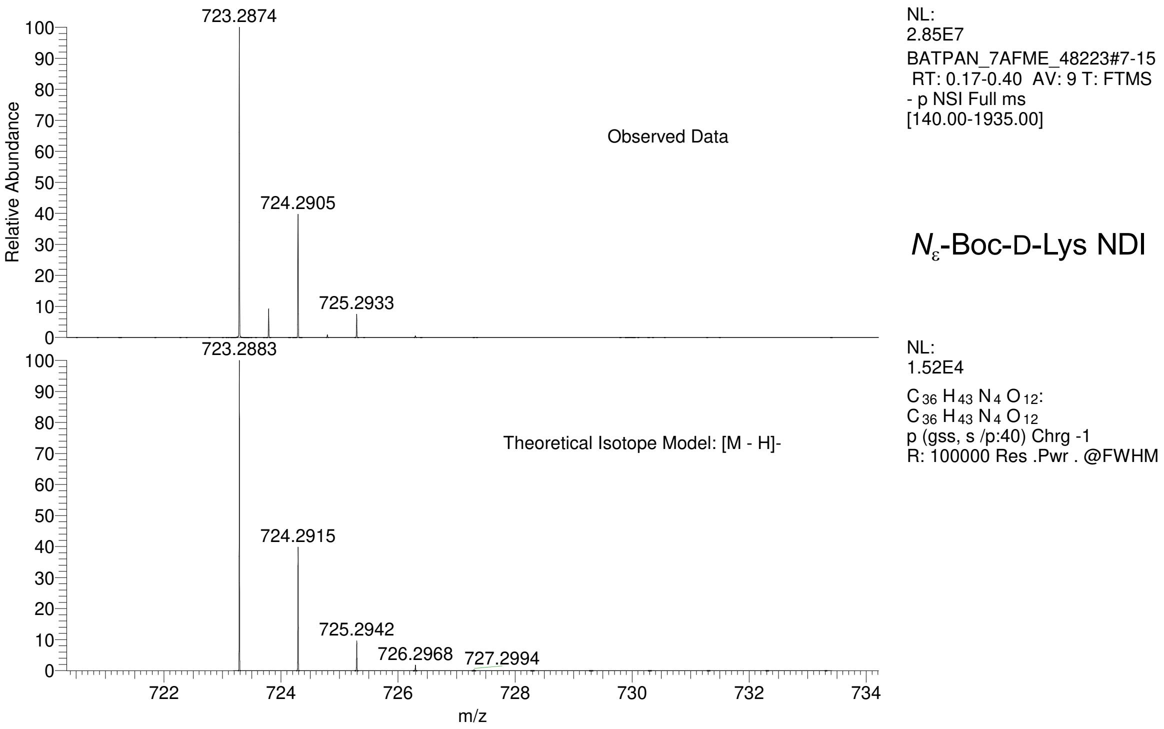

N_ε-Boc-D-Lys NDI

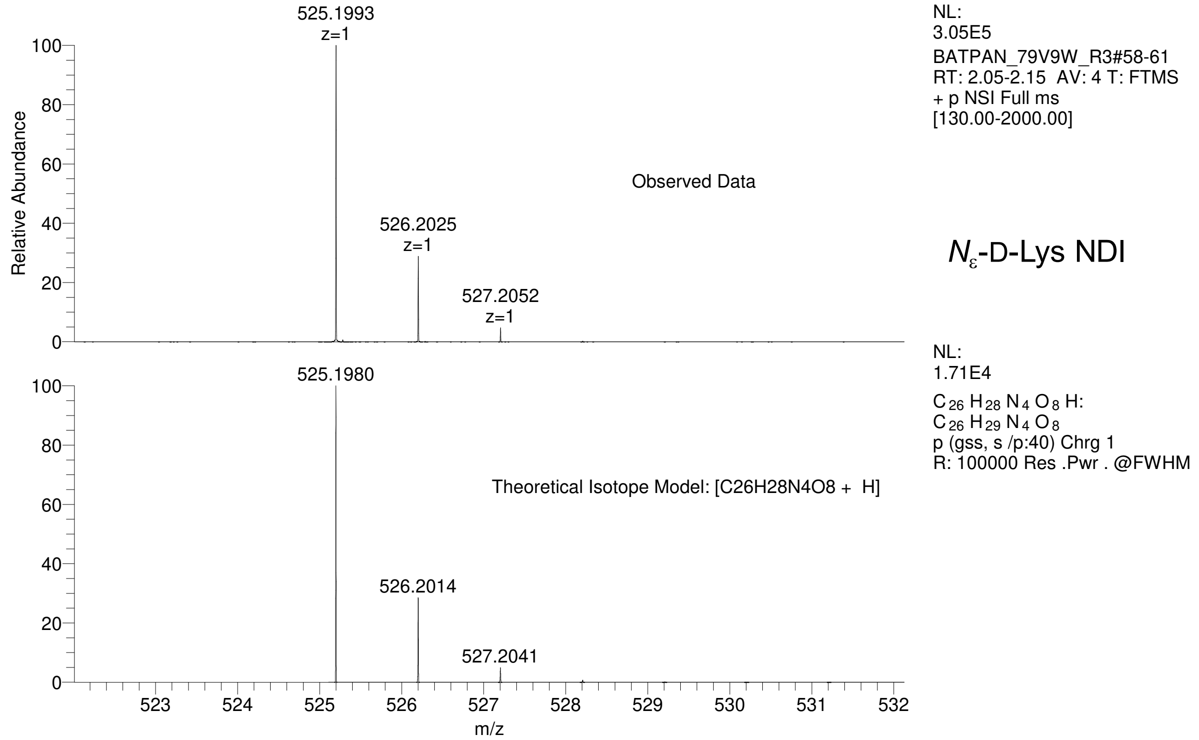

N_{ε}-D-Lys NDI

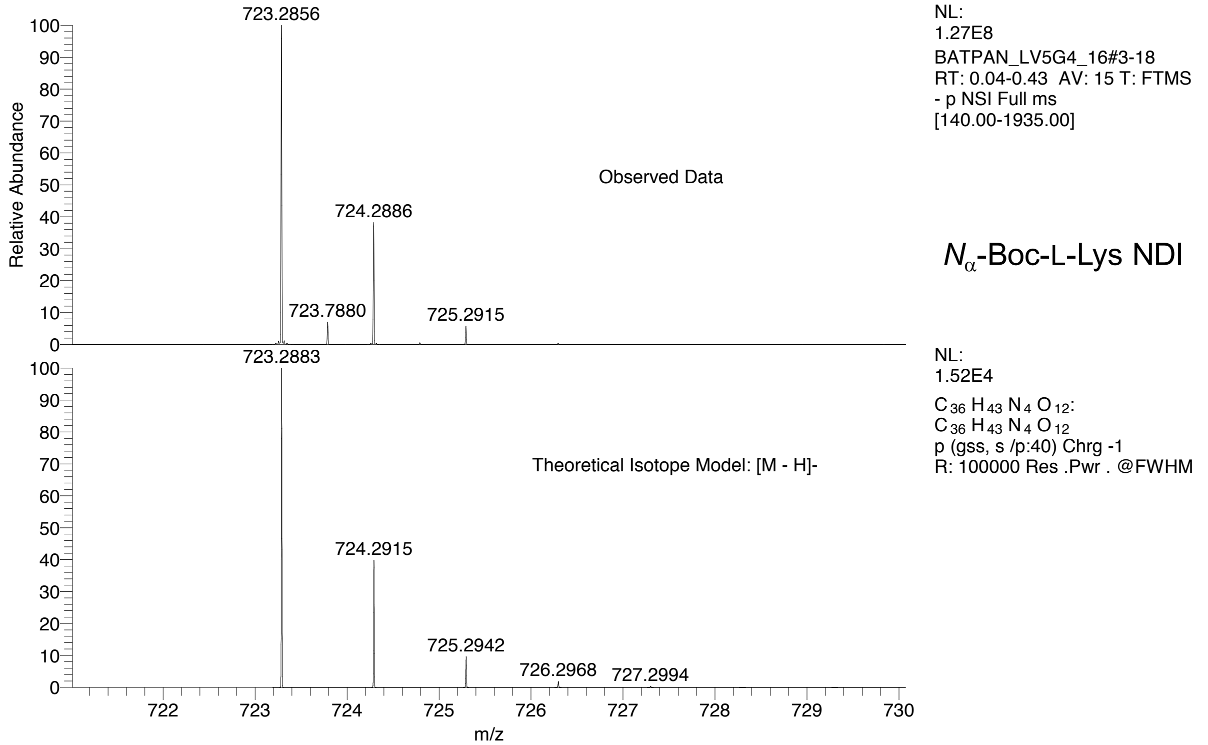

N_{α}-Boc-L-Lys NDI

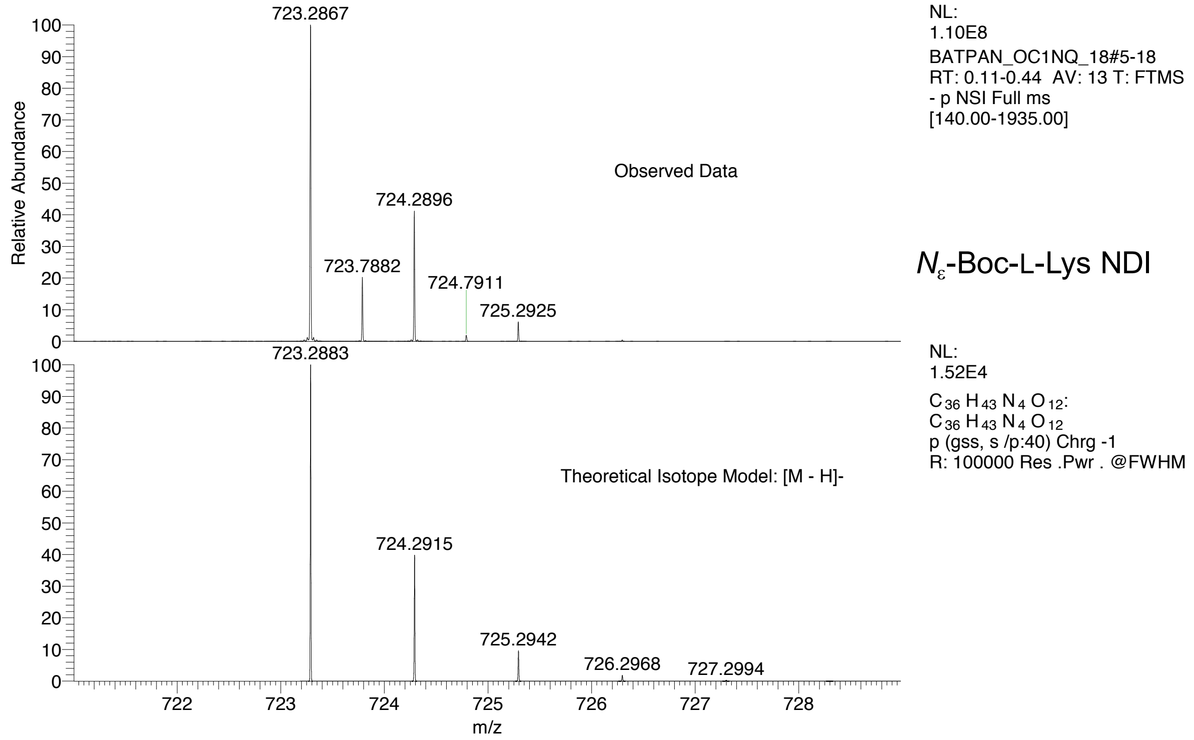

N$_{\varepsilon}$-Boc-L-Lys NDI

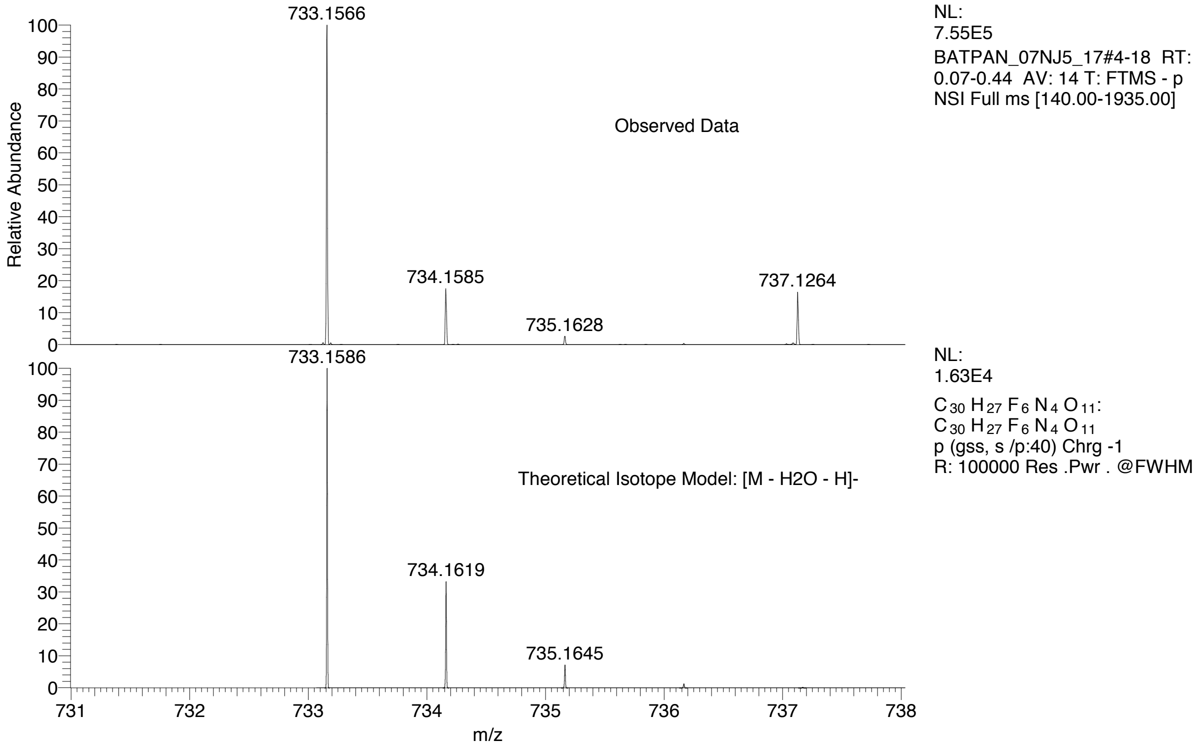

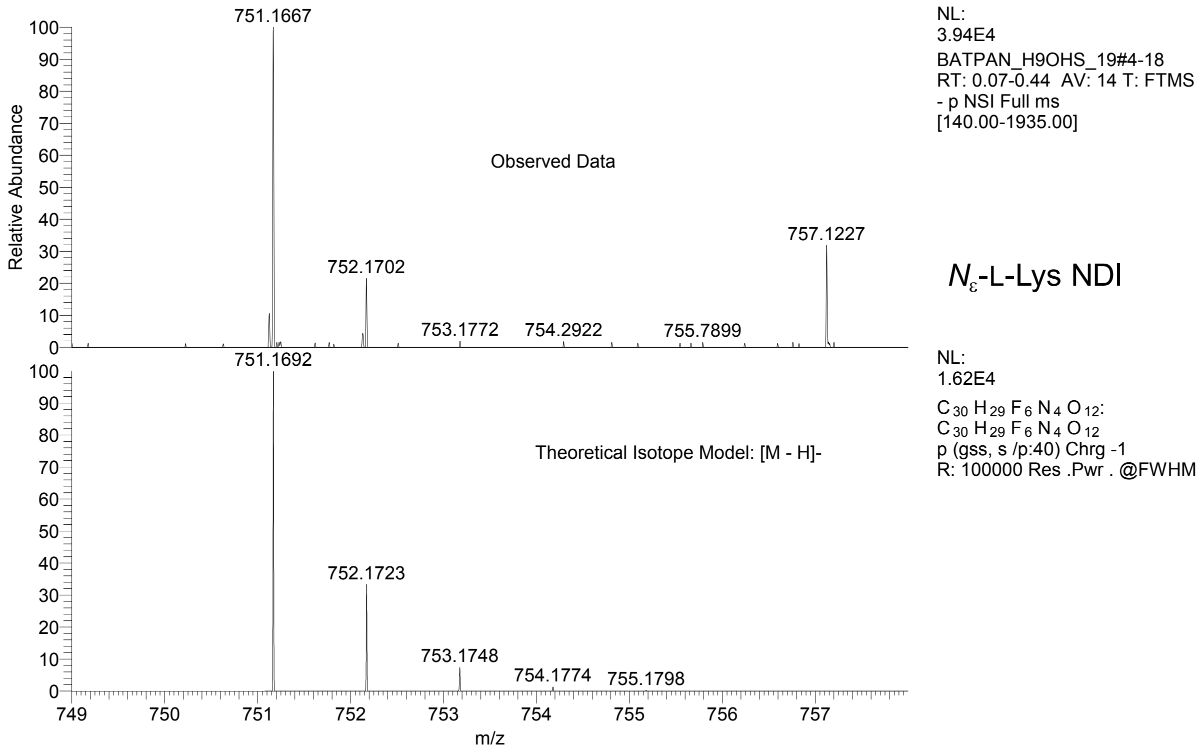

2. Conformation analysis

The G-quadruplex DNA sequences used in this study adopt either parallel (c-KIT2 and k-RAS) or hybrid (c-Kit1, BCL-2 and h-TELO) conformations in the presence of K^+, as determined by CD spectroscopy (spectra shown in **Figure S1**). **Figure S2** illustrates the overlaid CD spectra of all sequences. An in-depth discussion about the structural features (including the far-UV region) of these G-quadruplexes under our experimental conditions is provided in our previously reported work [1].

The CD thermal melting experiments were performed at single wavelength (spectra given in Section 3 in this document). However, it is important to test whether the DNA sequences used in this study maintain their conformation upon addition of NDI ligands. For this, CD spectra of all DNA sequences as well as DNA with ligands at the same concentrations as used for VTs have been recorded before the melting experiments (**Figures S3 – S9**). We have observed no change in conformation type in any of the cases; however, the deprotected ligands (mainly N_ε-Lys NDI) tend to aggregate, thus contribution to CD spectra above 300 nm may be observed. The data was collected at 23 °C in 10 mM potassium phosphate buffer, pH 7.4, and 100 mM potassium fluoride.

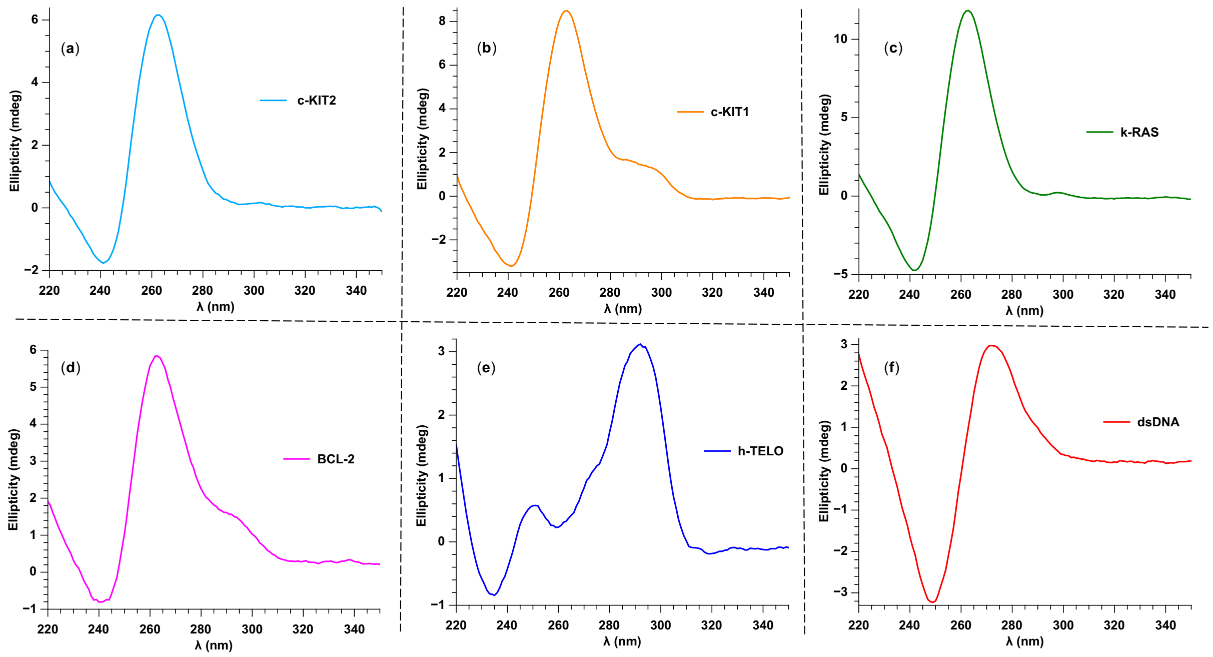

Figure S1. CD spectra of G-quadruples DNA sequences as well as dsDNA used in this study: (**a**) parallel c-KIT2 (5.3 μM), (**b**) hybrid c-KIT1 (5.2 μM), (**c**) parallel k-RAS (11.4 μM), (**d**) hybrid BCL-2 (10.8 μM), (**e**) hybrid h-TELO (9.8 μM), (**f**) dsDNA (10.5 μM). Data collected at 23 °C in 10 mM potassium phosphate buffer, pH 7.4, and 100 mM potassium fluoride.

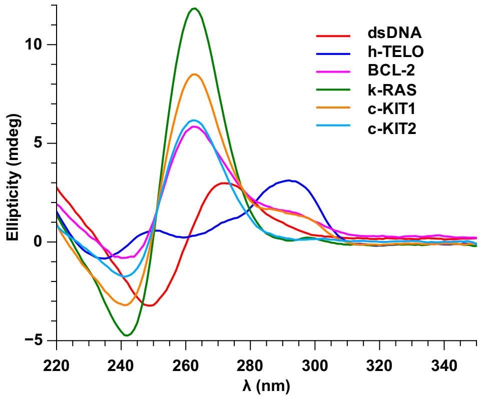

Figure S2. Overlaid CD spectra of all types of DNA used in this study. Concentrations are the same as given in the previous figure caption.

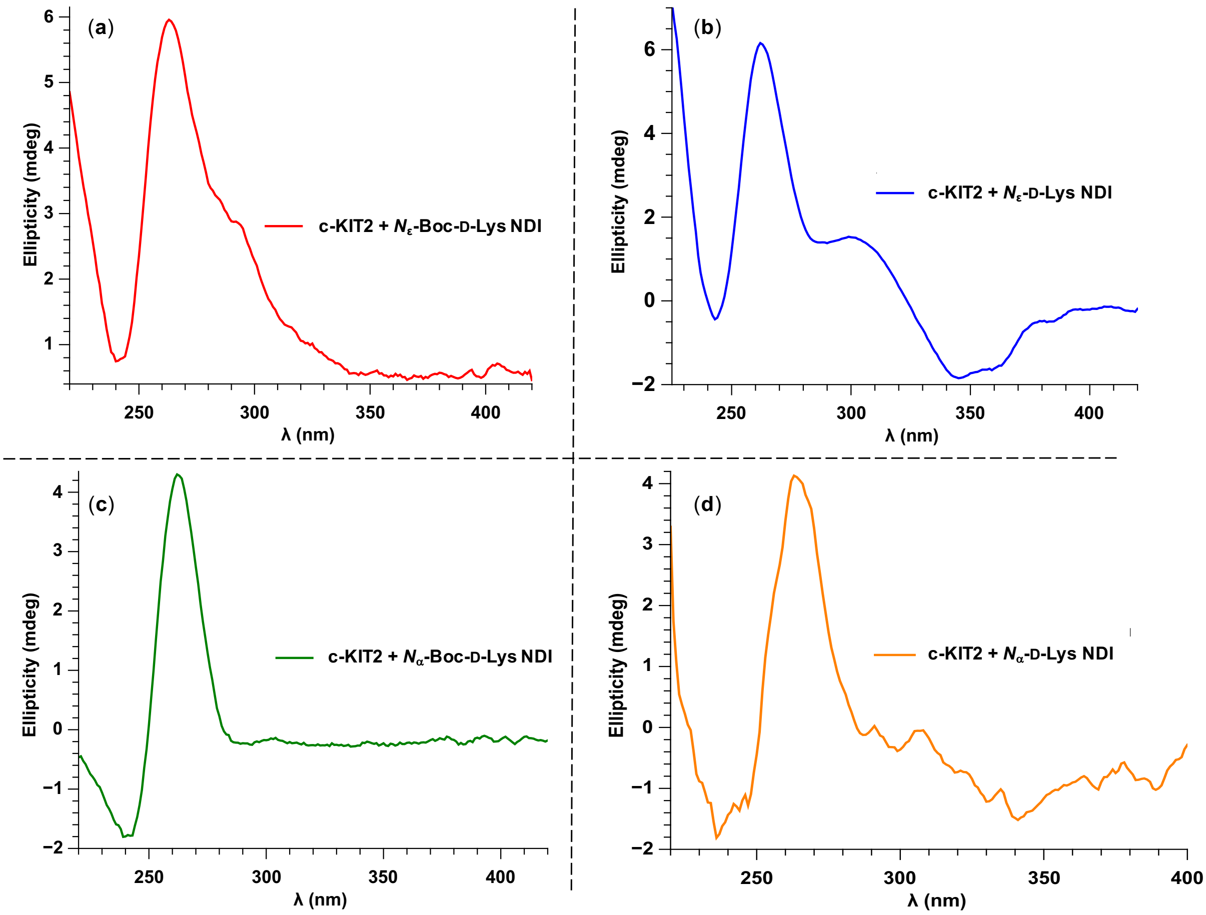

Figure S3. The CD spectra of c-KIT2 sequence interacting with: (**a**) ***N*ε-Boc-D-Lys NDI**, (**b**) ***N*ε-D-Lys NDI**, (**c**) ***N*α-Boc-D-Lys NDI**, (**d**) ***N*α-D-Lys NDI** before the VT experiments. Concentrations: the same as for VT studies.

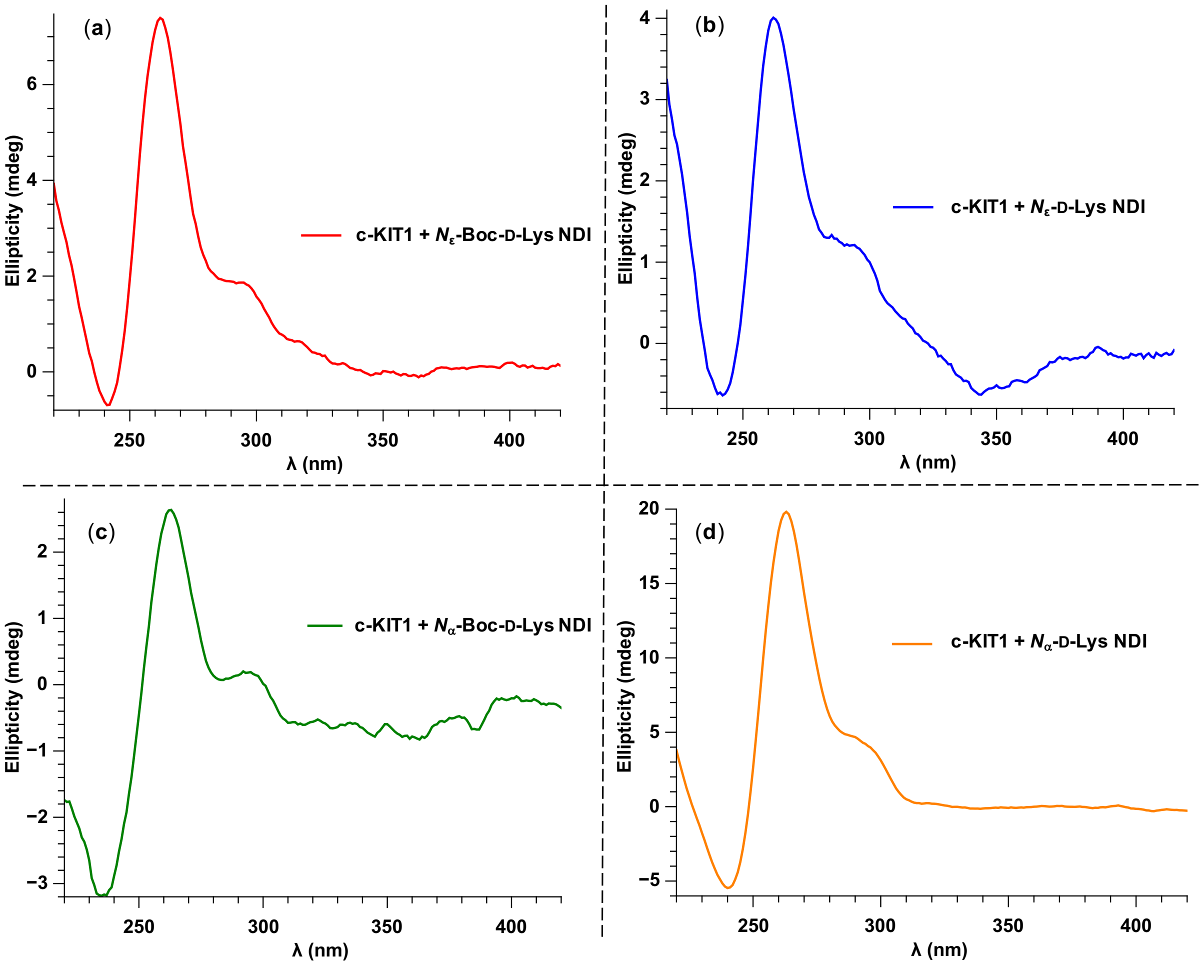

Figure S4. The CD spectra of c-KIT1 sequence interacting with: (**a**) ***N*$_ε$-Boc-D-Lys NDI**, (**b**) ***N*$_ε$-D-Lys NDI**, (**c**) ***N*$_α$-Boc-D-Lys NDI**, (**d**) ***N*$_α$-D-Lys NDI** before the VT experiments. Concentrations: the same as for VT studies.

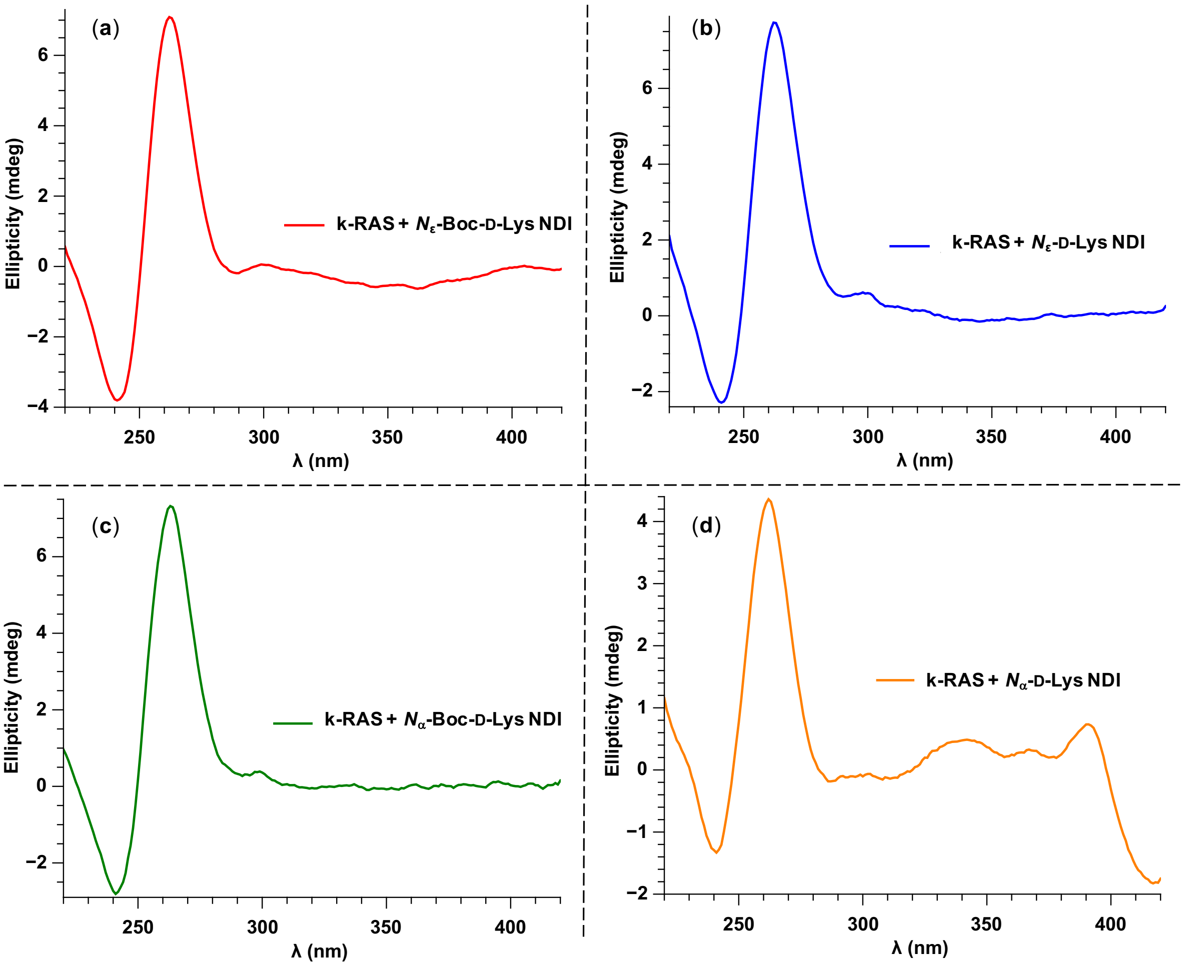

Figure S5. The CD spectra of k-RAS sequence interacting with: (**a**) ***N*ε-Boc-D-Lys NDI**, (**b**) ***N*ε-D-Lys NDI**, (**c**) ***N*α-Boc-D-Lys NDI**, (**d**) ***N*α-D-Lys NDI** before the VT experiments. Concentrations: the same as for VT studies.

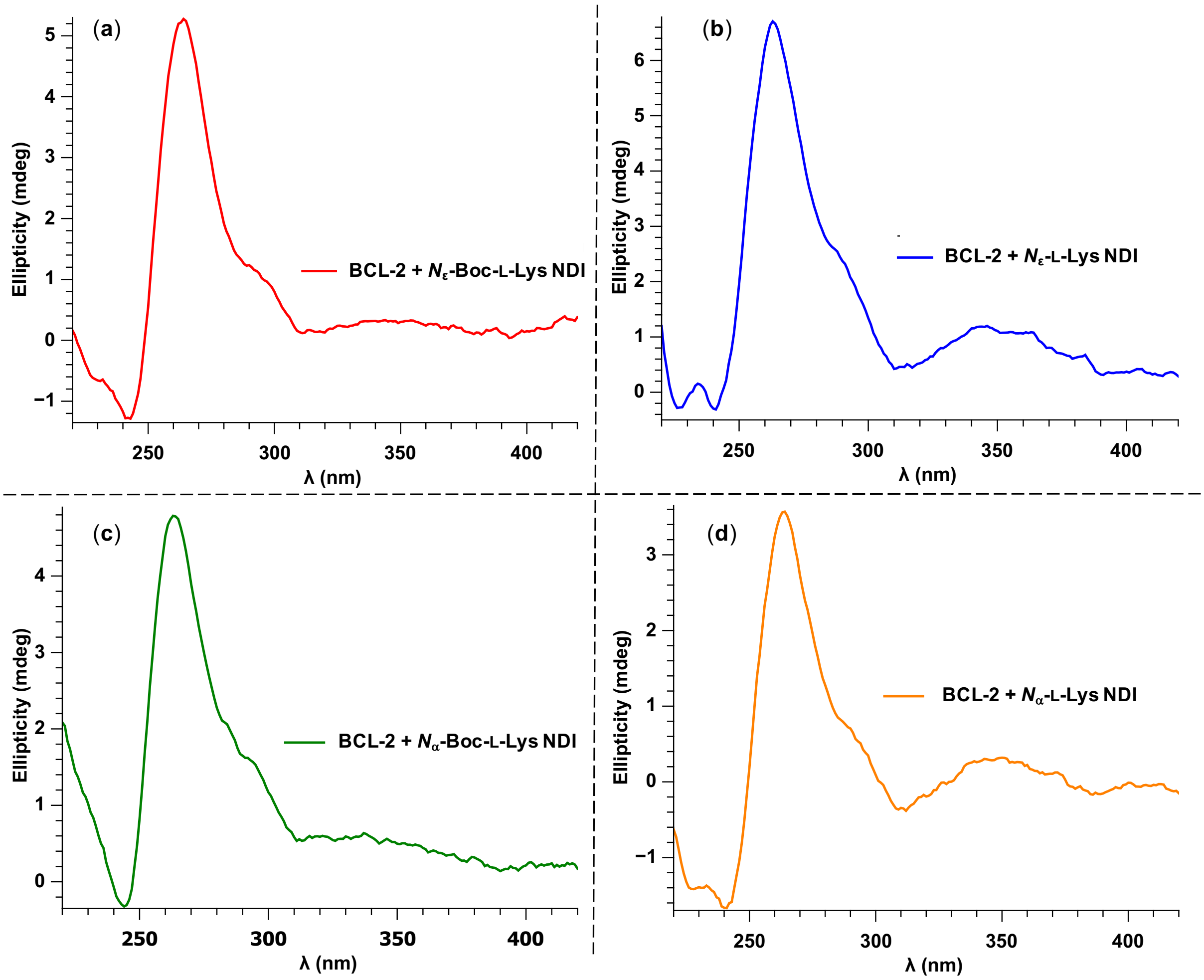

Figure S6. The CD spectra of BCL-2 sequence interacting with: (**a**) ***N*$_\varepsilon$-Boc-L-Lys NDI**, (**b**) ***N*$_\varepsilon$-L-Lys NDI**, (**c**) ***N*$_\alpha$-Boc-L-Lys NDI**, (**d**) ***N*$_\alpha$-L-Lys NDI** before the VT experiments. Concentrations: the same as for VT studies.

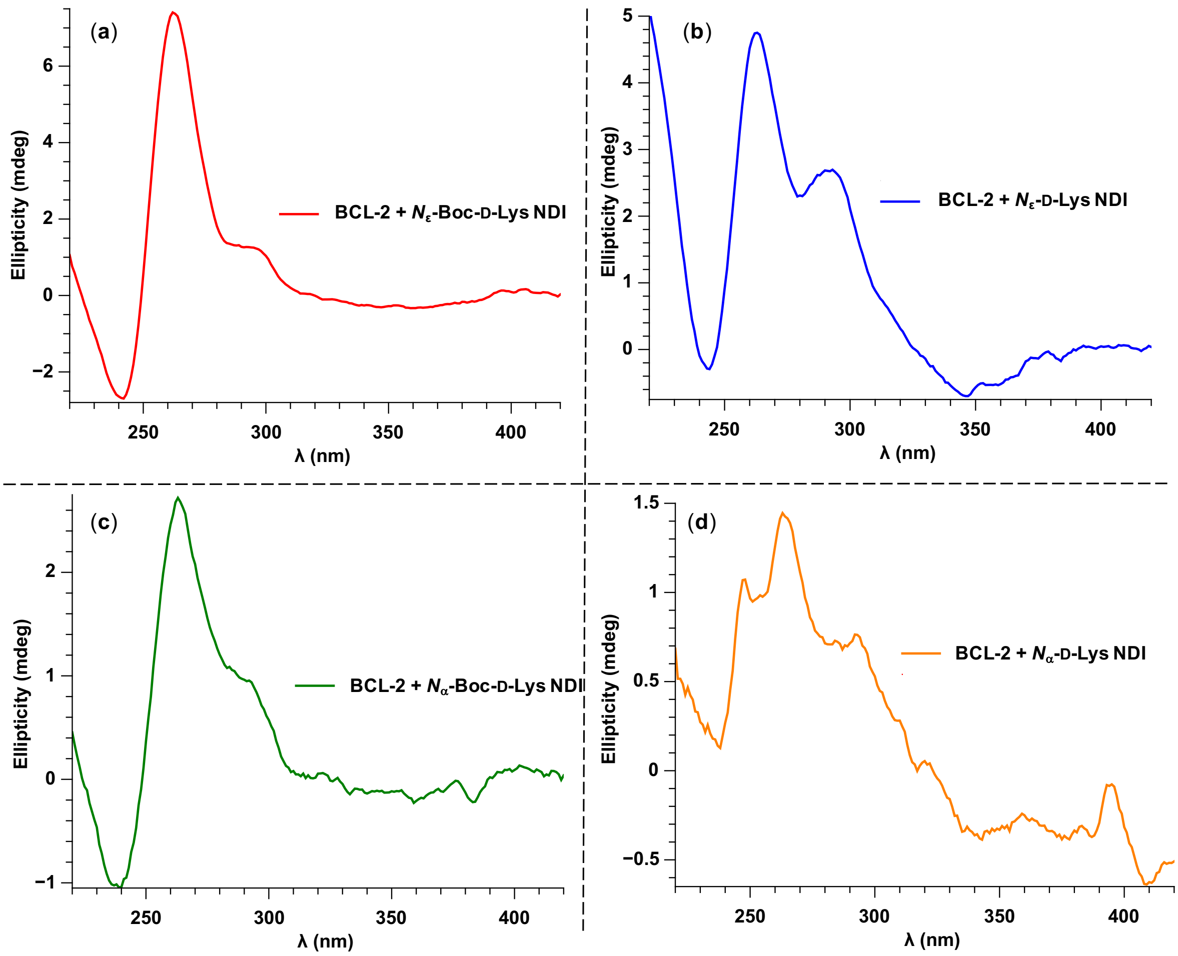

Figure S7. The CD spectra of BCL-2 sequence interacting with: (**a**) ***$N_ε$*-Boc-D-Lys NDI**, (**b**) ***$N_ε$*-D-Lys NDI**, (**c**) ***$N_α$*-Boc-D-Lys NDI**, (**d**) ***$N_α$*-D-Lys NDI** before the VT experiments. Concentrations: the same as for VT studies.

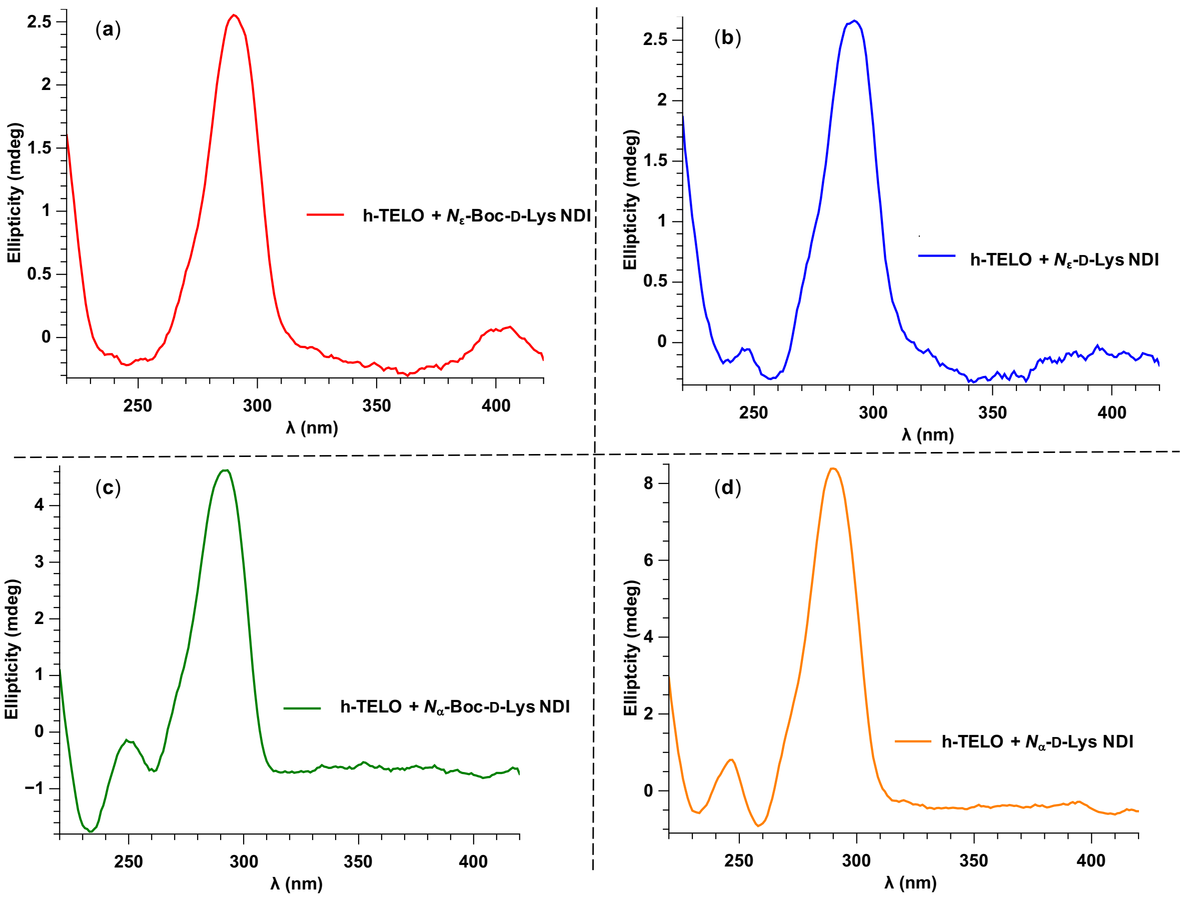

Figure S8. The CD spectra of h-TELO sequence interacting with: (**a**) ***N*$_\varepsilon$-Boc-D-Lys NDI**, (**b**) ***N*$_\varepsilon$-D-Lys NDI**, (**c**) ***N*$_\alpha$-Boc-D-Lys NDI**, (**d**) ***N*$_\alpha$-D-Lys NDI** before the VT experiments. Concentrations: the same as for VT studies.

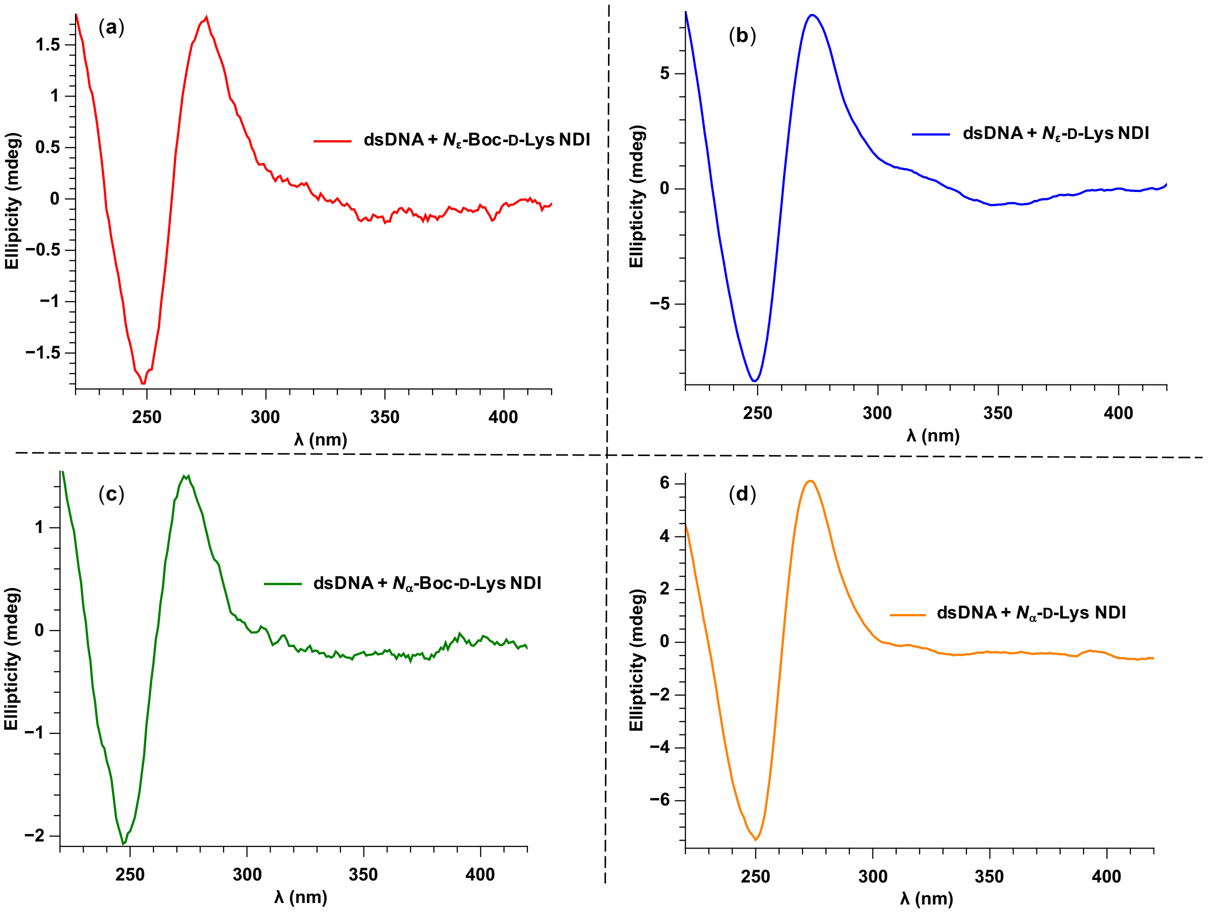

Figure S9. The CD spectra of dsDNA sequence interacting with: (**a**) ***N*$_\varepsilon$-Boc-D-Lys NDI**, (**b**) ***N*$_\varepsilon$-D-Lys NDI**, (**c**) ***N*$_\alpha$-Boc-D-Lys NDI**, (**d**) ***N*$_\alpha$-D-Lys NDI** before the VT experiments. Concentrations: the same as for VT studies.

All DNA sequences are expected to unfold and denaturate and the end of melting experiments. To show this, examples of VT spectra of each type of conformation (parallel and hybrid) for the DNA by itself and the DNA with ligand across a broad wavelength range are shown in **Figure S10**. These indicate no change in conformation throughout the melting cycle (*i.e.* as the temperature is increased), but just DNA denaturation. A complete series of VT CD spectra across broad wavelength range for DNA sequences and L-NDIs are provided in previously reported work [1]. The samples containing DNA-D-NDIs behave in an identical way (*i.e.* denaturation as the temperature is increased).

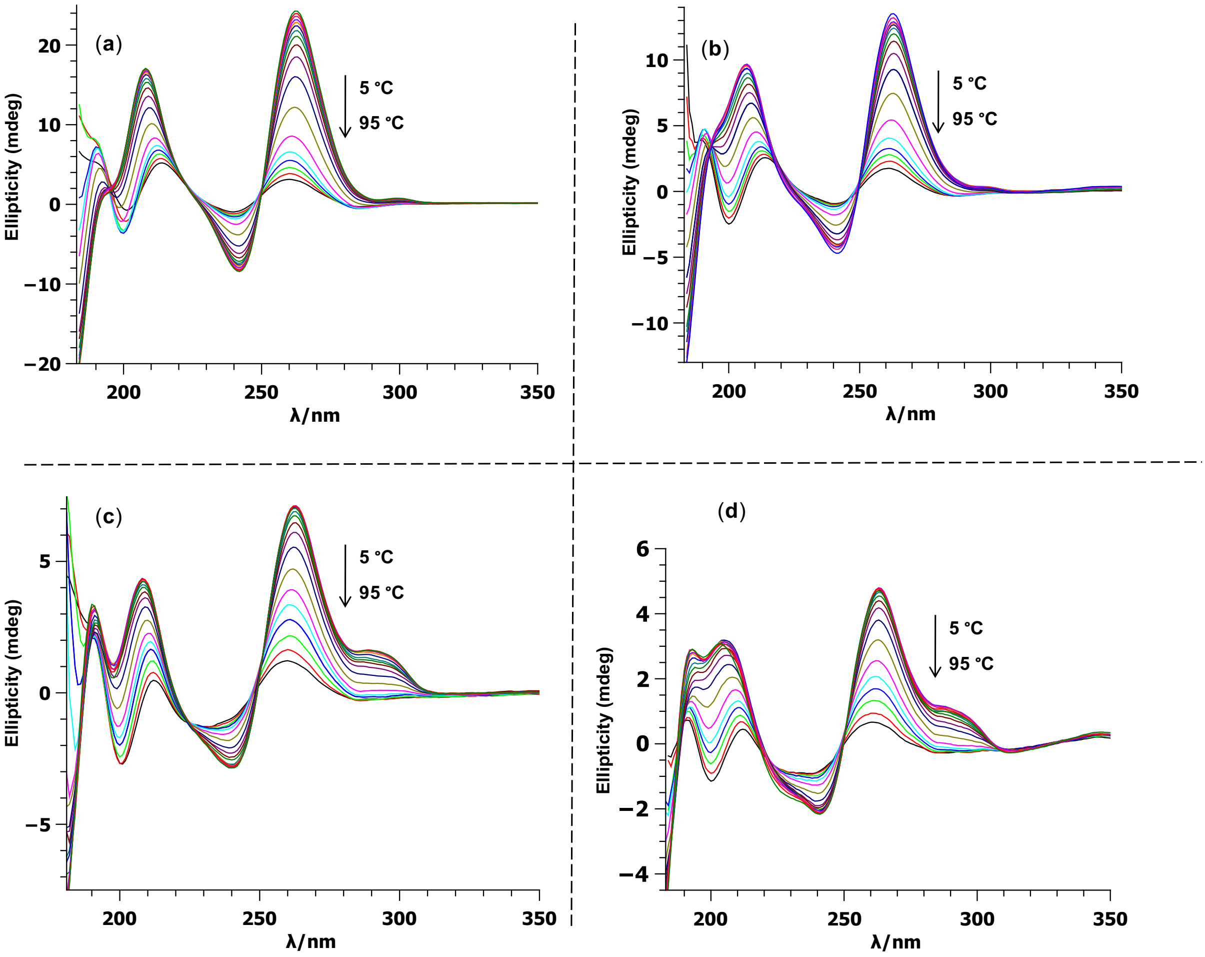

Figure S10. The CD melting associated spectra of (**a**) parallel k-RAS by itself (20 μM) and (**b**) its assembly formed with ***N*$_\varepsilon$-Boc-L-Lys NDI**; (**c**) hybrid c-KIT1 by itself (10 μM) and (**d**) its assembly formed with ***N*$_\varepsilon$-Boc-L-Lys NDI**.

To further show that the G-quadruplex DNAs used in this study do not change their conformation upon ligand binding, titrations of increasing equivalents (equiv) of NDI into DNA (0 to 10 equiv) were performed. We provide CD spectra collected at 23 °C for sequences assembling into parallel quadruplex (c-KIT2) as well as hybrid conformations of the oncogene promoter c-KIT1 and telomeric h-TELO (**Figure S11**). No change in conformation was observed at increasing equivalents of NDI, leading to the conclusion that these G-quadruplexes do not change their topologies upon ligand interaction.

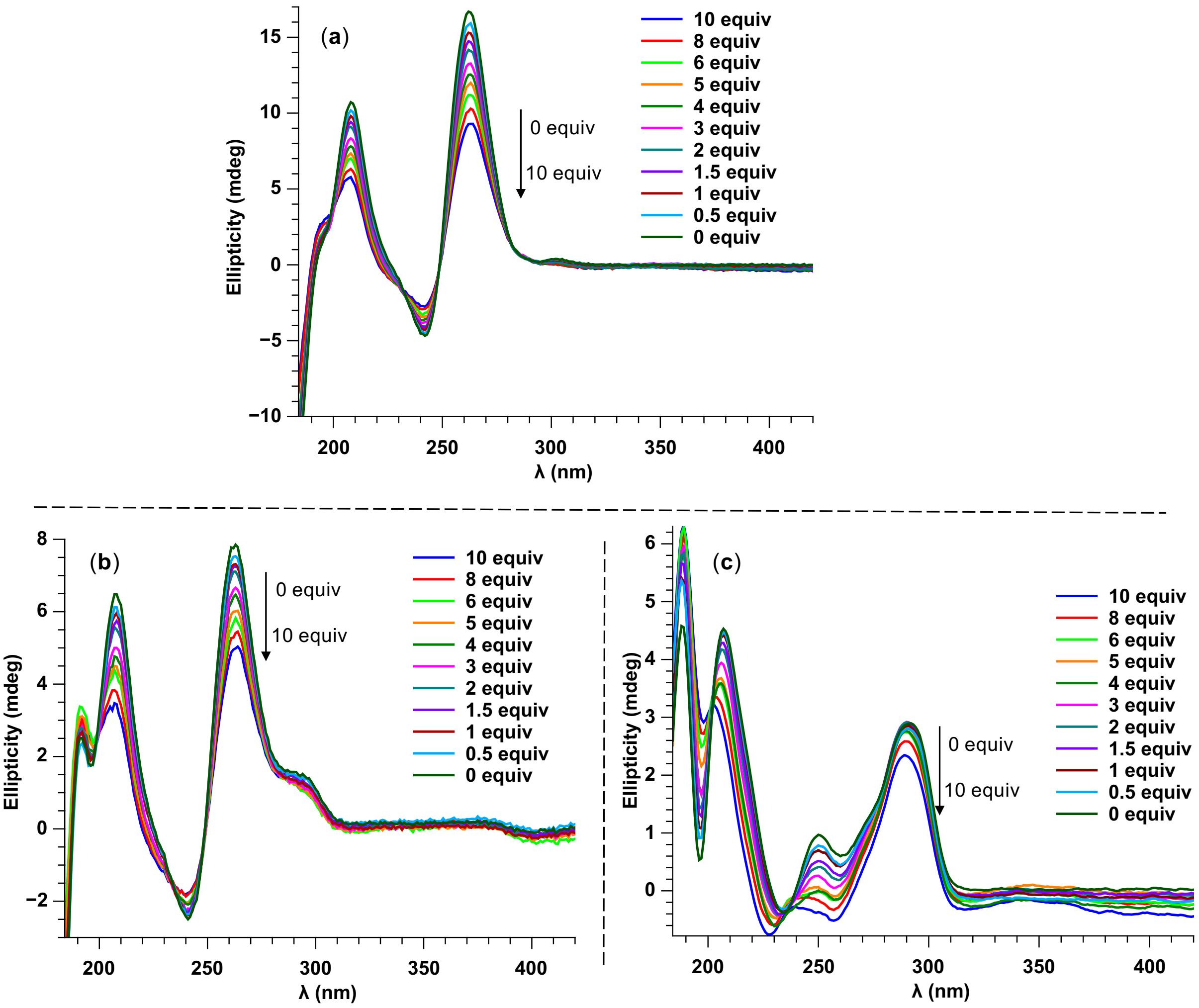

Figure S11. The CD associated spectra at 23 °C of (**a**) parallel oncogene promoter c-KIT2, (**b**) hybrid oncogene promoter c-KIT1, and (**c**) hybrid telomeric sequence h-TELO upon titration of increasing equivalents (0 – 10 equiv) of ***N_{ε}*-L-Lys NDI**.

A 5-point smoothing was applied to all graphs shown in this Section when processed.

3. CD thermal melting studies

The VT CD spectra and Boltzmann fitting curves (shown in red) performed at single-wavelength (*i.e.* λ_{max} in each case) are provided in **Figures S12 – S18**. Ligand solutions were prepared at concentrations of 300 μM for all NDIs, with the exception of ***N_{α}*-D-Lys NDI**, which was prepared at 75 μM because of the aggregation issue. The ligand was added as 10 equivalents to the DNA solution for VT CD studies. The

concentration of DNA used for each experiment and λ_{max} at which the experiment was acquired are also given in the corresponding figures. Conversion of ellipticity to molar ellipticity allowed for direct comparison of the T_m obtained. All the data was collected in 10 mM PBS and 100 mM potassium fluoride.

3.1. c-KIT2

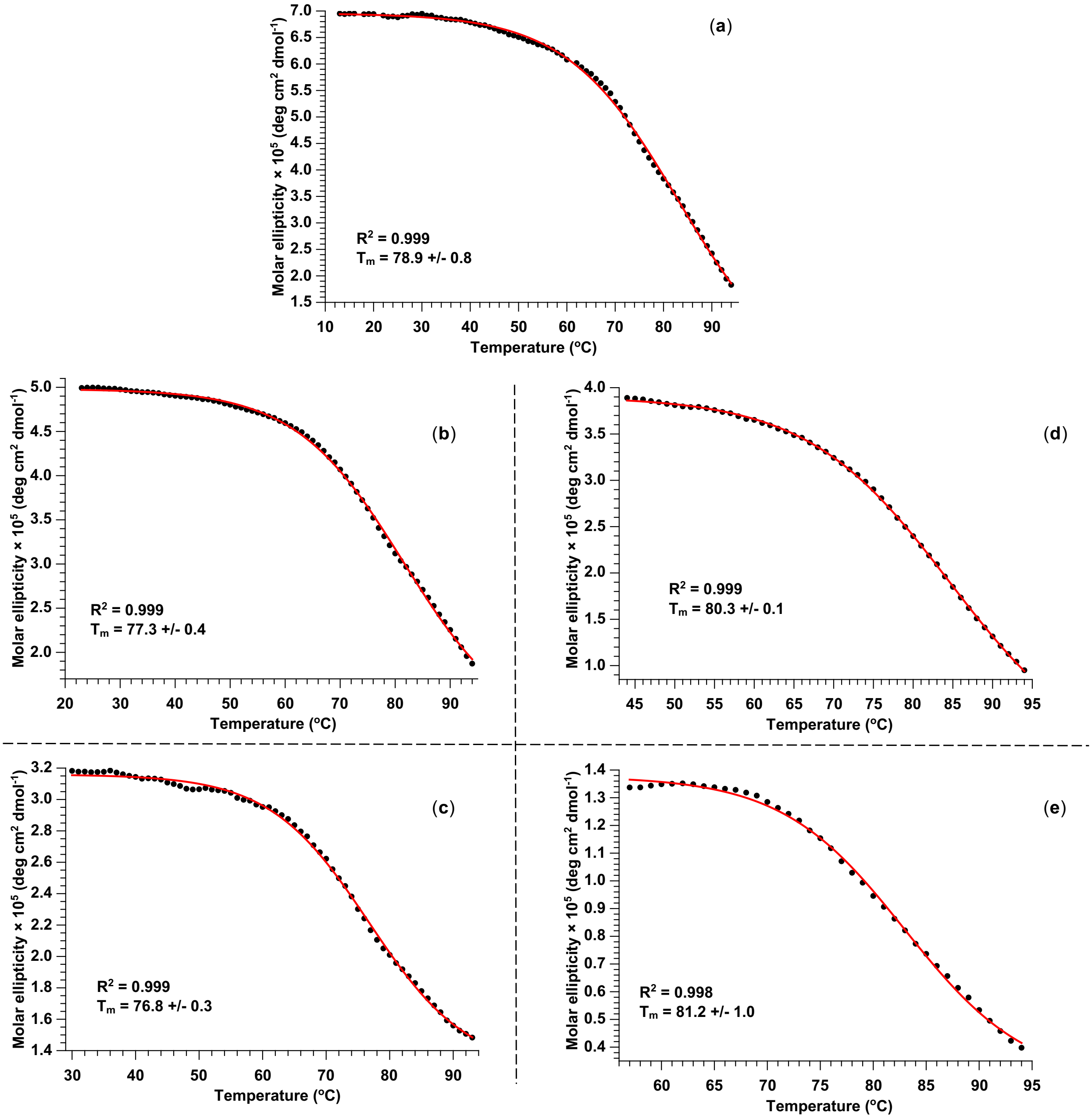

Figure S12. The VT CD spectra associated with the melting of 5.3 μM c-KIT2 by itself at 262 nm (**a**); 5.2 μM c-KIT2 with ***N*****ε-Boc-D-Lys NDI** at 262 nm (**b**); 3.4 μM c-

KIT2 with ***N***$\boldsymbol{N_\varepsilon}$**-D-Lys NDI** at 262 nm (**c**); 5.7 μM c-KIT2 with $\boldsymbol{N_\alpha}$**-Boc-D-Lys NDI** at 264 nm (**d**); 5.7 μM c-KIT2 with $\boldsymbol{N_\alpha}$**-D-Lys NDI** at 264 nm (**e**).

3.2. c-KIT1

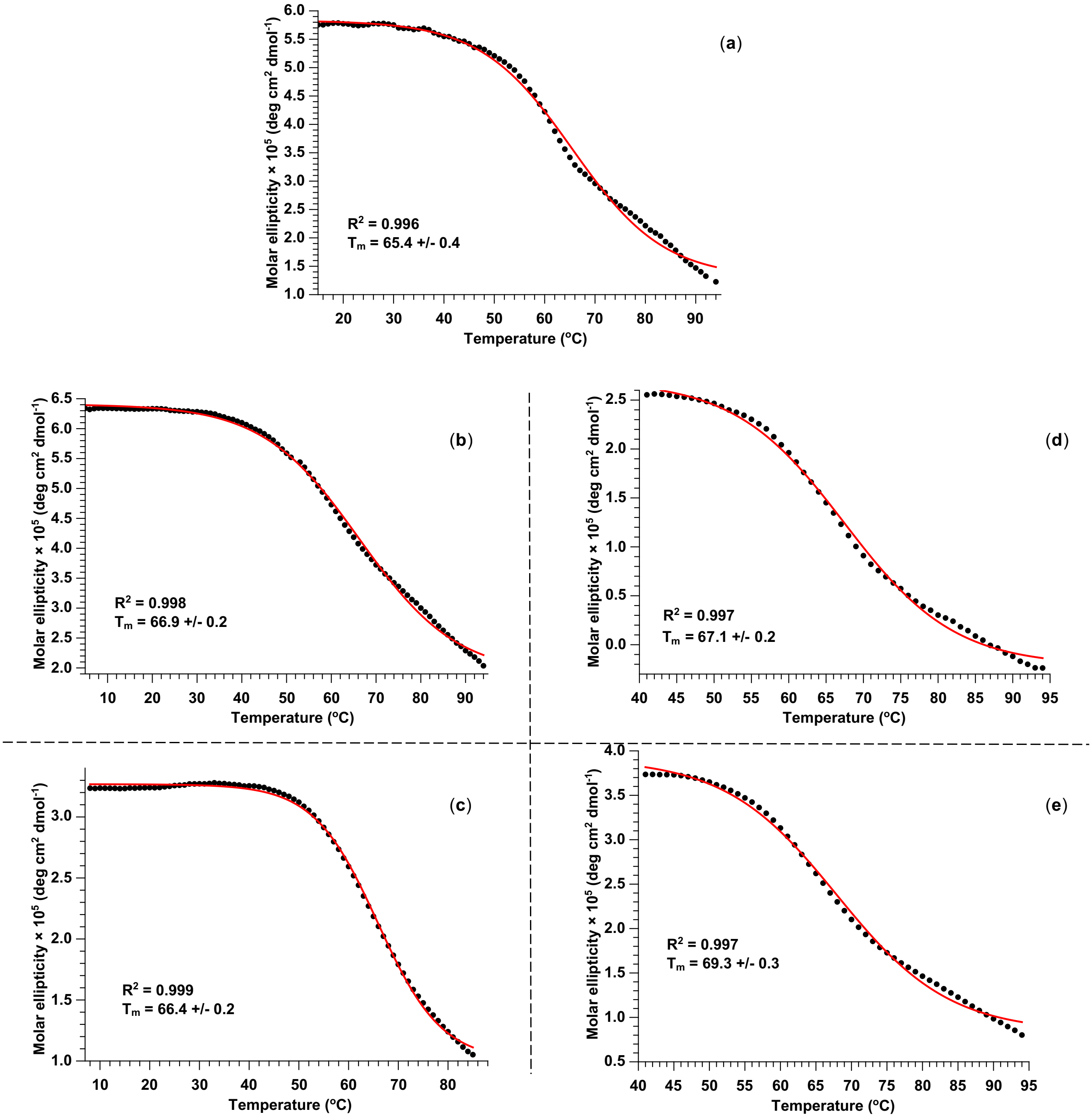

Figure S13. The VT CD spectra associated with the melting of 5.2 μM c-KIT1 by itself at 262 nm (**a**); 11.5 μM c-KIT1 with $\boldsymbol{N_\varepsilon}$**-Boc-D-Lys NDI** at 263 nm (**b**); 5.1 μM c-KIT1 with $\boldsymbol{N_\varepsilon}$**-D-Lys NDI** at 262 nm (**c**); 5.2 μM c-KIT1 with $\boldsymbol{N_\alpha}$**-Boc-D-Lys NDI** at 263 nm (**d**); 11.2 μM c-KIT1 with $\boldsymbol{N_\alpha}$**-D-Lys NDI** at 262 nm (**e**).

3.3. k-RAS

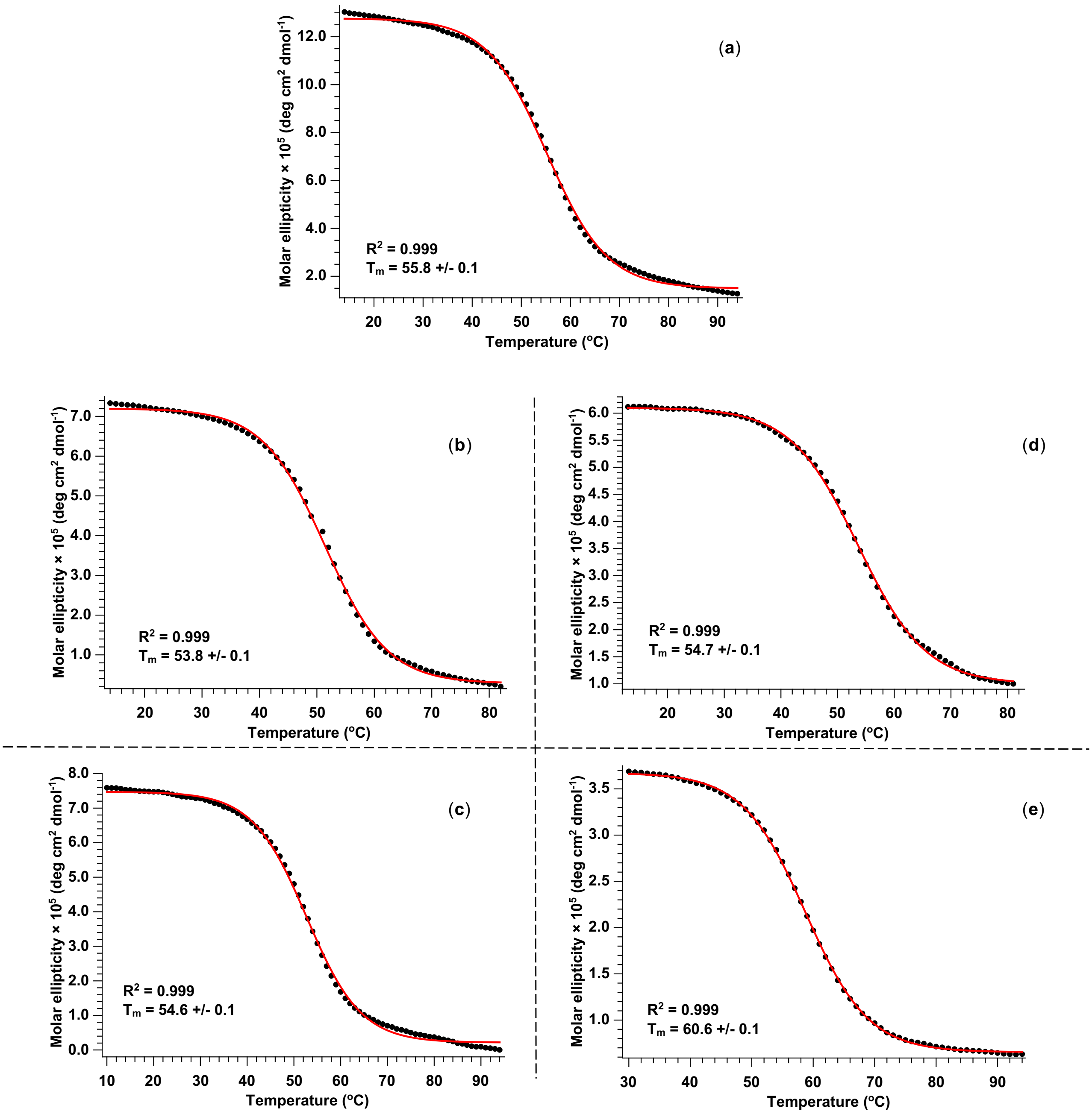

Figure S14. The VT CD spectra associated with the melting of 11.4 μM k-RAS by itself at 262 nm (**a**); 10.0 μM k-RAS with **N_{ε}-Boc-D-Lys NDI** at 262 nm (**b**); 10.0 μM k-RAS with **N_{ε}-D-Lys NDI** at 263 nm (**c**); 11.4 μM k-RAS with **N_{α}-Boc-D-Lys NDI** at 262 nm (**d**); 9.9 μM k-RAS with **N_{α}-D-Lys NDI** at 260 nm (**e**).

3.4. BCL-2

3.4.1. BCL-2 with L-NDIs

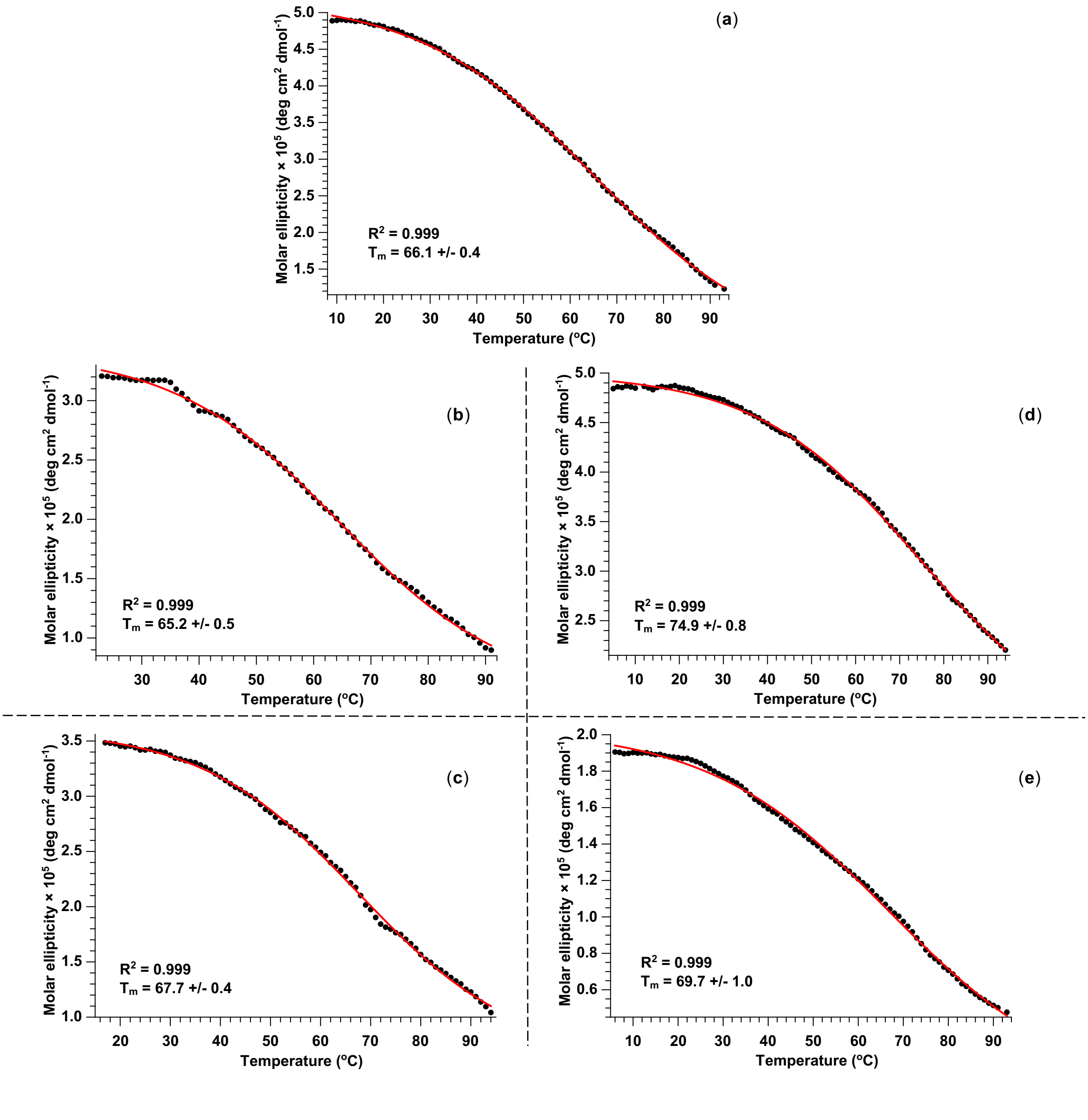

Figure S15. The VT CD spectra associated with the melting of 10.8 μM BCL-2 by itself at 262 nm (**a**); 10.8 μM BCL-2 with ***N*$_\varepsilon$-Boc-L-Lys NDI** at 262 nm (**b**); 10.8 μM BCL-2 with ***N*$_\varepsilon$-L-Lys NDI** at 262 nm (**c**); 10.8 μM BCL-2 with ***N*$_\alpha$-Boc-L-Lys NDI** at 263 nm (**d**); 9.8 μM BCL-2 with ***N*$_\alpha$-L-Lys NDI** at 263 nm (**e**).

3.4.2. BCL-2 with D-NDIs

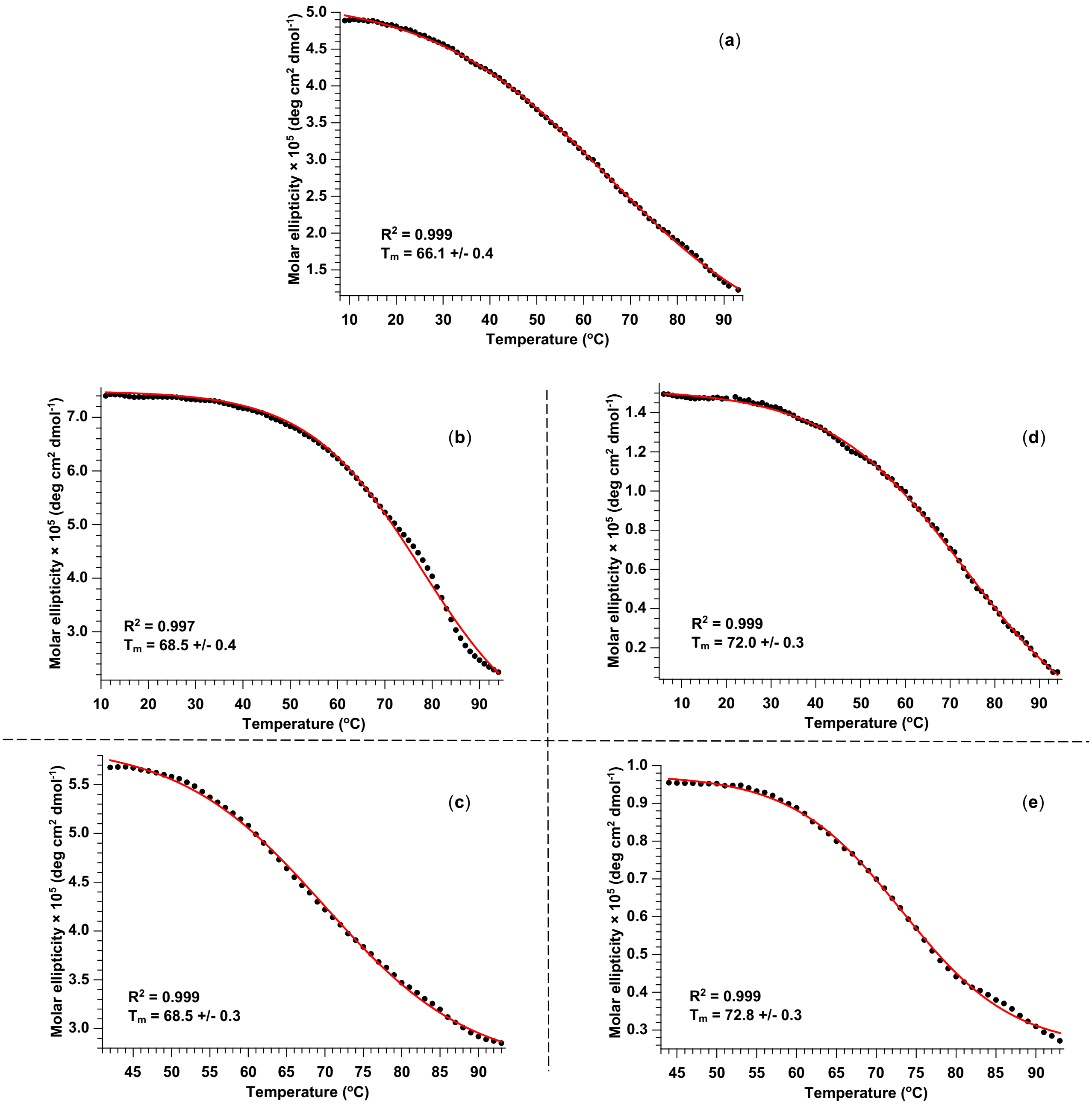

Figure S16. The VT CD spectra associated with the melting of 10.8 μM BCL-2 by itself at 262 nm (**a**); 10.6 μM BCL-2 with ***N*ε-Boc-D-Lys NDI** at 262 nm (**b**); 4.4 μM BCL-2 with ***N*ε-D-Lys NDI** at 263 nm (**c**); 5.0 μM BCL-2 with ***N*α-Boc-D-Lys NDI** at 262 nm (**d**); 3.0 μM BCL-2 with ***N*α-D-Lys NDI** at 263 nm (**e**).

3.5. h-TELO

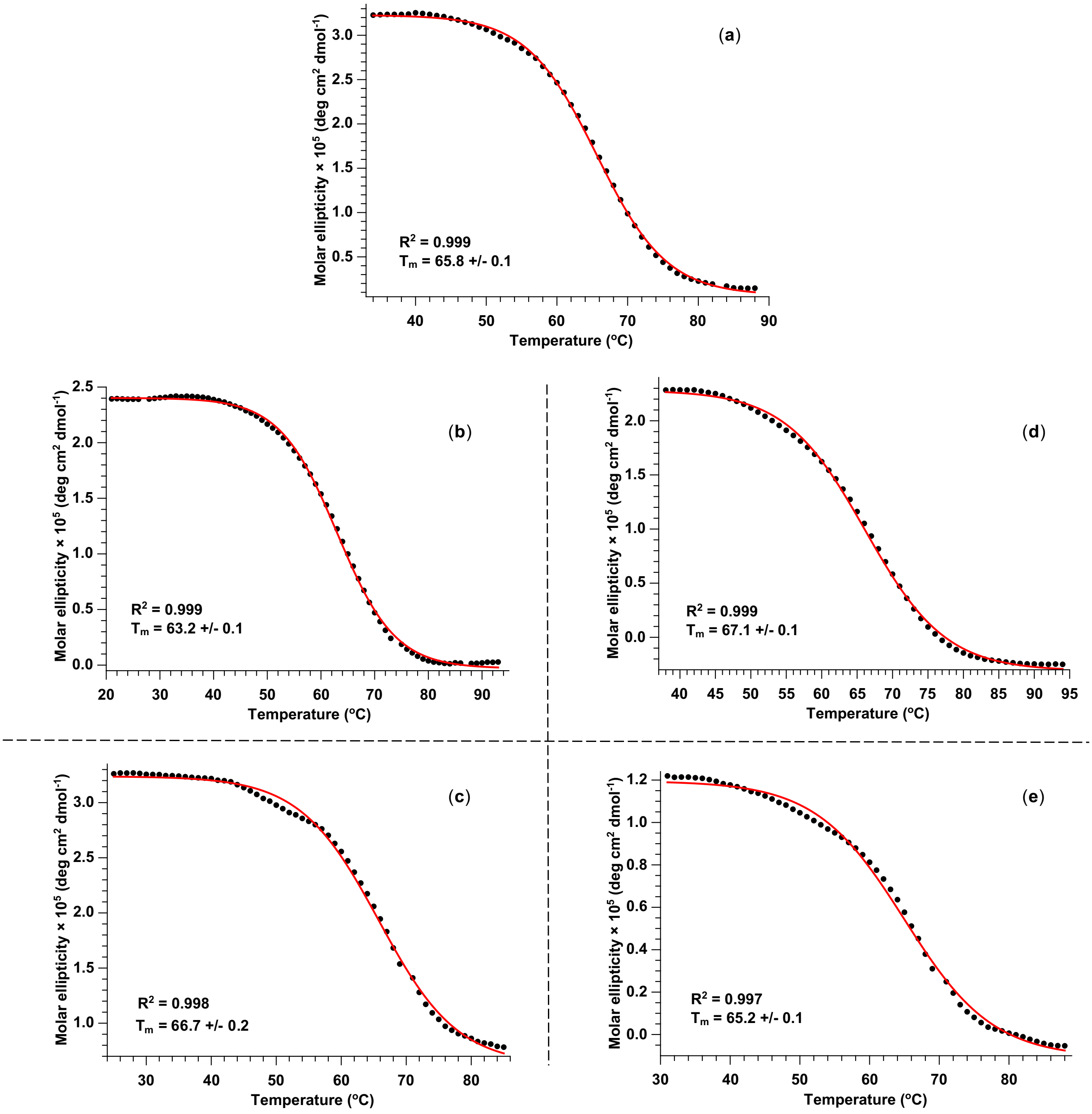

Figure S17. The VT CD spectra associated with the melting of 9.8 μM h-TELO by itself at 290 nm (**a**); 9.8 μM h-TELO with **N_{ε}-Boc-D-Lys NDI** at 293 nm (**b**); 9.8 μM h-TELO with **N_{ε}-D-Lys NDI** at 292 nm (**c**); 10.3 μM h-TELO with **N_{α}-Boc-D-Lys NDI** at 292 nm (**d**); 10.4 μM h-TELO with **N_{α}-D-Lys NDI** at 290 nm (**e**).

3.6. dsDNA

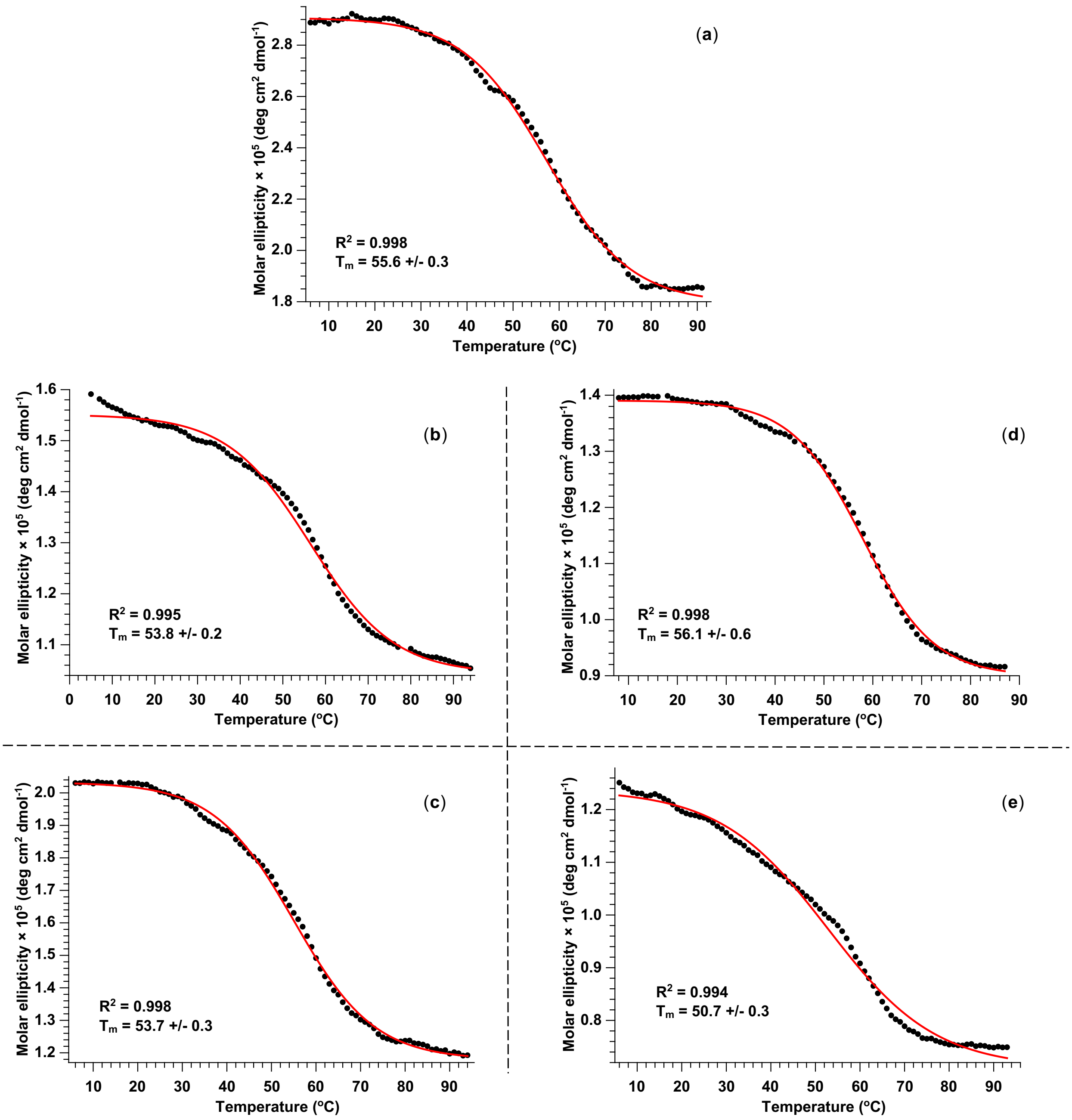

Figure S18. The VT CD spectra associated with the melting of 10.5 μM dsDNA by itself at 272 nm (**a**); 10.0 μM dsDNA with ***N*ε-Boc-D-Lys NDI** at 273 nm (**b**); 10.1 μM dsDNA with ***N*ε-D-Lys NDI** at 273 nm (**c**); 10.0 μM dsDNA with ***N*α-Boc-D-Lys NDI** at 272 nm (**d**); 10.0 μM dsDNA with ***N*α-D-Lys NDI** at 273 nm (**e**).

4. Size-exclusion chromatography analysis

The purity of G-quadruplex DNA sequences used in this study was achieved through standard desalting method, as certified by the manufacturer. This purity level is recommended by the manufacturer for most of the applications. However, all sequences were analysed by size-exclusion chromatography[2] (SEC) to further asses their purity and type of secondary structures formed. The SEC profiles of sequences used in this study are provided in **Figure S19**. The sequences used are mostly in monomeric form.

The data was acquired using an Agilent 1100 HPLC instrument with single-wavelength detector. Experimental conditions are given below:

Sample preparation: the DNA was annealed under the same conditions as for (VT) CD studies (*i.e.* 10 mM phosphate buffer and 100 mM potassium fluoride). Each sample was filtered using a PTFE 0.2 μm filter. The samples were not further diluted; due to a variation in the starting concentrations, different injection volumes were calculated for each DNA sequence in order to achieve optimum resolution (see Table S1). The concentrations used for SEC analysis are in the range of those used for VTCD experiments.

Column: BioSep-SEC-S 2000 (Phenomenex), dimensions: 30 × 0.46 cm, particle size: 5 μm, pore size: 150 Å.

Analytical method:

Flow rate: 0.35 mL/min

Temperature: 22 °C

Elution profile: isocratic 0.1 M KH_2PO_4/K_2HPO_4 pH 7.1

Monitored wavelength: 260 nm

Injection volume: depending on the sequence concentration (see Table S1)

Table S1. Concentration of each sequence used for SEC studies and the corresponding injection volume used in each case.

Type of sequence	Concentration of sequence (μM)	Injection volume (μL)
c-KIT2	20.3*	1.3
c-KIT1	18.2	1.4
k-RAS	10.4	2.6
BCL-2	3.3	7.9
h-TELO	12.6	2.1
dsDNA	10.0	5.0

*Due to high concentration of this sequence, dimer is also possible to form in this case.

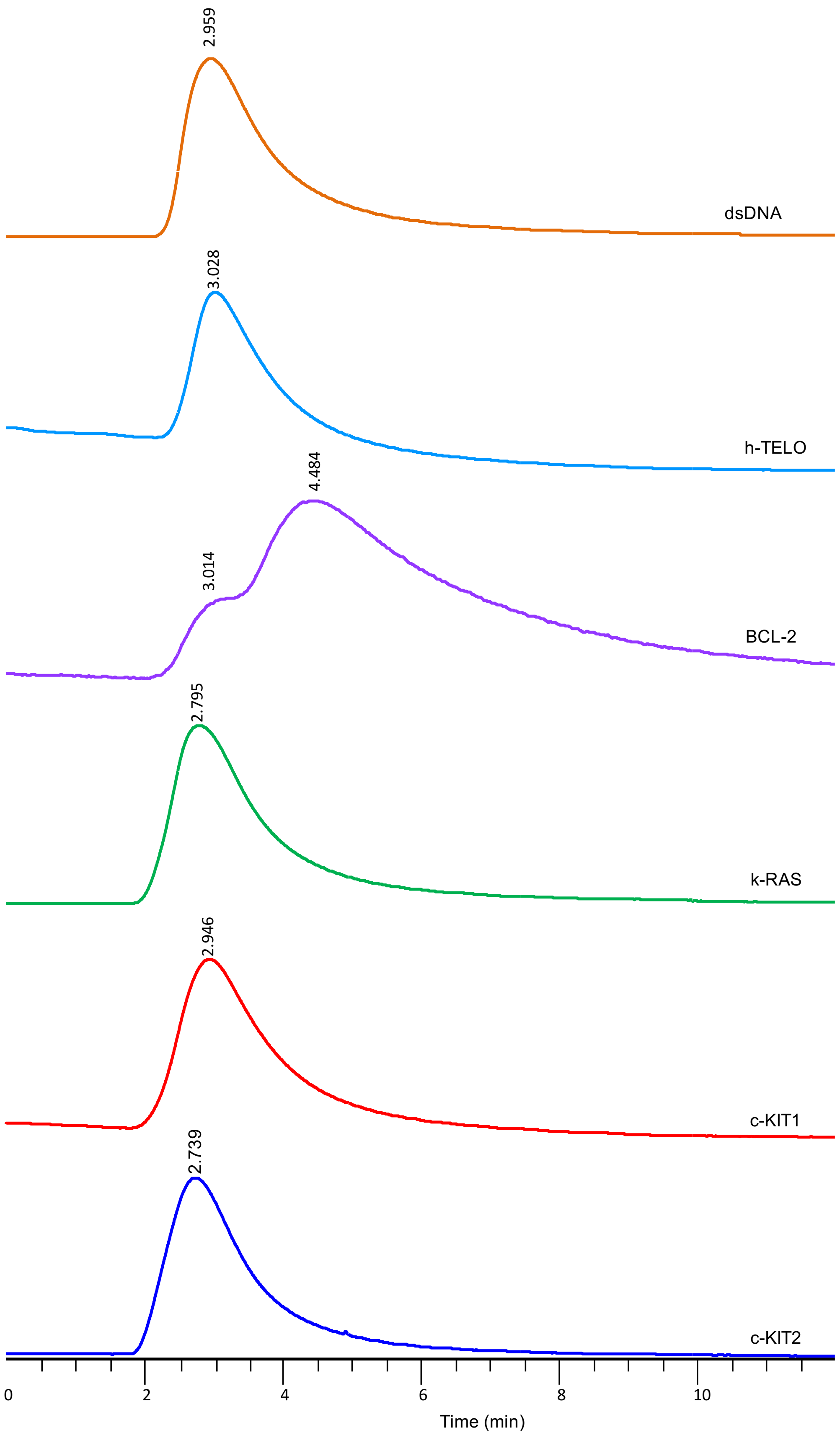

Figure S19. SEC profiles of sequences used in this study.

5. References

1. Răsădean, D.M.; Sheng, B.; Dash, J.; Pantoş, G.D. Amino-Acid-Derived Naphthalenediimides as Versatile G-Quadruplex Binders. *Chemistry - A European Journal* **2017**, *23*, 8491–8499.
2. Benabou, S.; Eritja, R.; Gargallo, R. Variable-Temperature Size Exclusion Chromatography for the Study of the Structural Changes in G-Quadruplex. *ISRN Biochemistry* **2013**, *2013*, 1–7.
